# Dynamic contrast-enhanced MRI radiomics nomogram for predicting axillary lymph node metastasis in breast cancer

**DOI:** 10.1186/s40644-022-00450-w

**Published:** 2022-04-04

**Authors:** Deling Song, Fei Yang, Yujiao Zhang, Yazhe Guo, Yingwu Qu, Xiaochen Zhang, Yuexiang Zhu, Shujun Cui

**Affiliations:** 1 Graduate Faculty, Hebei North University, 12 Changqing Road, Qiaoxi District, Zhangjiakou, 075000 China; 2 Department of Radiology, The First Affiliated Hospital of Wenzhou Medical University, Nanbaixiang New District, Ouhai District, Wenzhou, 32000 Zhejiang China; 3 Department of Radiology, The First Affiliated Hospital of Hebei North University, 12 Changqing Road, Qiaoxi District, Zhangjiakou, 075000 China

**Keywords:** Breast cancer, Axillary lymph node metastasis, Radiomics, Preoperative prediction

## Abstract

**Purpose:**

The goal of this study is to develop and validate a radiomics nomogram integrating the radiomics features from DCE-MRI and clinical factors for the preoperative diagnosis of axillary lymph node (ALN) metastasis in breast cancer patients.

**Procedures:**

A total of 432 patients with breast cancer were enrolled in this retrospective study and divided into a training cohort (*n* = 296) and a validation cohort (*n* = 136). Radiomics features were extracted from the second phase of dynamic contrast enhanced (DCE) MRI images. The least absolute shrinkage and selection operator (LASSO) regression method was used to screen optimal features and construct a radiomics signature in the training cohort. Multivariable logistic regression analysis was used to establish a radiomics nomogram model based on the radiomics signature and clinical factors. The predictive performance of the nomogram was quantified with respect to discrimination and calibration, which was further evaluated in the independent validation cohort.

**Results:**

Fourteen ALN metastasis-related features were selected to construct the radiomics signature, with an area under the curve (AUC) of 0.847 and 0.805 in the training and validation cohorts, respectively. The nomogram was established by incorporating the histological grade, multifocality, MRI report lymph node status and radiomics signature and showed good calibration and excellent performance for ALN detection (AUC of 0.907 and 0.874 in the training and validation cohorts, respectively). The decision curve, which demonstrated the radiomics nomogram, displayed promising clinical utility.

**Conclusions:**

The radiomics nomogram can be used as a noninvasive and reliable tool to assist clinicians in accurately predicting ALN metastasis in breast cancer preoperatively.

**Supplementary Information:**

The online version contains supplementary material available at 10.1186/s40644-022-00450-w.

## Background

In recent years, greater numbers of younger patients have presented with breast cancer, with a mortality rate ranking first among malignant tumors in females [1, 2]. Clinical investigations have revealed that the 5-year survival rate is 98% for lymph node (LN)-negative breast cancer patients. For LN-positive patients, the survival rate drops to 84% [3]. Identifying axillary lymph node (ALN) status remains important because it is among the most influential diagnostic and prognostic factors.

Radical breast cancer plus axillary lymph node dissection (ALND) was once considered the standard treatment for breast cancer. However, more than 70% of women with early-stage breast cancer have no ALN metastasis [4], so any kind of axillary surgery can be regarded as overtreatment. Compared with ALND, sentinel lymph node biopsy (SLNB) is less invasive and has fewer complications since it only selectively removes the first draining lymph node from the tumor [5]. Although the rate of damage to blood vessels and nerves in SLNB is lower than that in ALND, shoulder dysfunction, numbness, nerve damage and lymphedema may still occur at an unacceptable frequency [6, 7]. Furthermore, the American College of Surgeons Oncology Group Z0011 trial confirmed that there was no significant effect on overall survival between SLNB alone and SLNB + ALND in patients with one or two SLN metastases. Both groups accepted breast-conserving surgery and systemic therapy. The results indicated that patients with one or two lymph node (LN) metastases should be classified as low risk, and they should not be directed to immediately undergo ALND [8]. Some studies have disputed the necessity of SLNB in evaluating ALN status preoperatively [9, 10], and the results of these reports suggested that SLNB or ALND should be selectively avoided, especially for low-risk SLN-positive patients.

Considering the above, if clinicians can preoperatively identify high-risk ALN metastases in breast cancer patients, then these patients may be suitable candidates for ALND. To date, several studies have attempted to establish models for evaluating ALN status based on pathological and genetic characteristics [11], such as lymphovascular invasion and serum miRNA, but these data are only available during surgery or after immunohistochemical examination, which may lead to inadequate clinical implications. Therefore, it is of great importance to develop a predictive tool that approximates SLNB for predicting the ALN status of breast cancer patients preoperatively.

Dynamic contrast-enhanced MRI (DCE-MRI) has been applied to discriminate between benign and malignant tumors, define tumor sizes and detect occult lesions. Kim et al. [12] attempted to predict ALN metastasis with lymph node characteristics, such as the long axis, short axis, and cortical thickness of the lymph node. These predictors showed moderate prediction efficacy and discrimination power with AUCs of 0.730, 0.670 and 0.773, and low sensitivity leads to missed diagnoses in some breast cancer patients with ALN metastasis [13–15]. Radiomics is an image quantitative feature data mining technology based on the high-throughput extraction of rich, deep image features of lesions to establish correlations between image features and clinical information, which can be used to improve the accuracy of tumor diagnosis, prognosis, and prediction [16–18]. Moreover, radiomics is also an important part of precision medicine and individualized treatment, especially in oncology [19, 20]. Previous studies have shown that the radiomics features of the primary tumor obtained from MRI images can be regarded as potential noninvasive biomarkers for the preoperative prediction of lymph node metastasis before surgery [21–23].

Here, we hypothesized that the radiomics features based on DCE-MRI sequences could predict ALN metastasis more accurately than the radiomics features based on other imaging modalities, and the purpose of our study was to establish and validate a nomogram based on DCE-MRI radiomics features and clinical factors for the preoperative prediction of ALN status in patients with breast cancer.

## Methods

### Patients

The Ethics Committee of The First Affiliated Hospital of Hebei North University approved this study, and, for this type of study, did not require informed consent. Approximately 553 patients who were confirmed to have breast cancer from April 2017 to October 2019 were retrospectively enrolled in this study. The inclusion criteria of the study were as follows: (1) patients with invasive breast carcinoma confirmed by biopsy or intraoperative resection, (2) patients who underwent DCE-MRI examination 1 week before surgery, and (3) all patients who received SLNB/ALND when SLNB was positive. The exclusion criteria were as follows: (1) preoperative treatment that included radiotherapy, chemotherapy and endocrine therapy; and (2) incomplete clinical data.

**Fig. 1:**
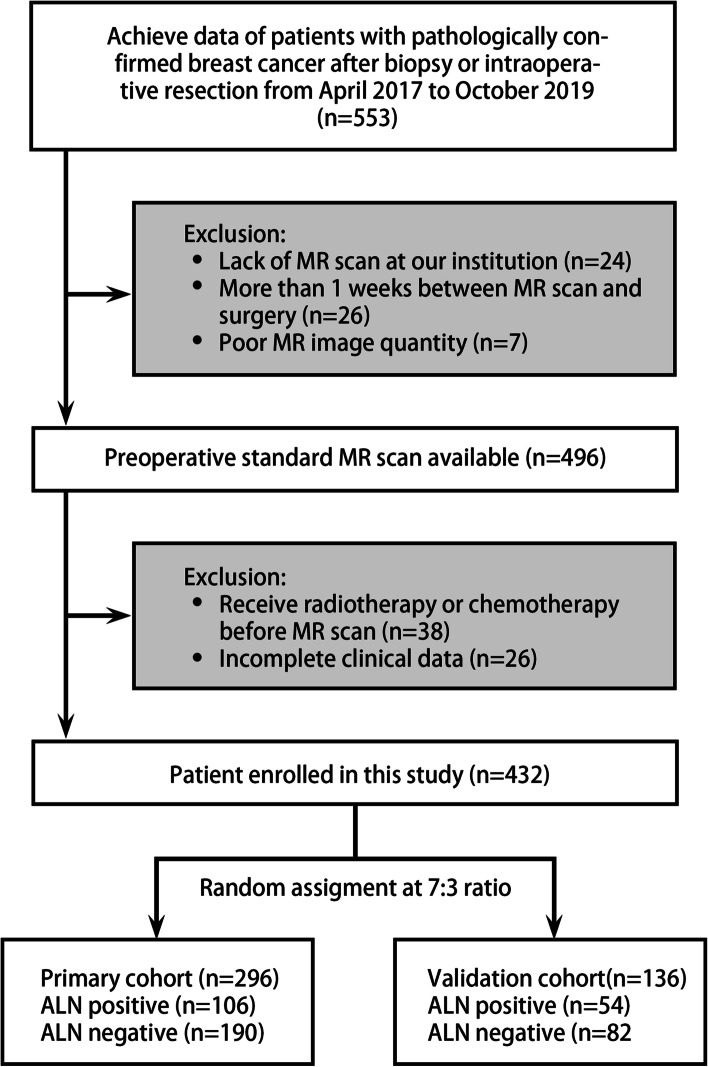
Flow chart of the study population, with exclusion criteria

In total, 432 patients were finally enrolled and divided into the training cohort (106 ALN-positive patients and 190 ALN-negative patients) and the validation cohort (54 ALN-positive patients and 82 ALN-negative patients) at a ratio of 7:3 (Fig. 1).

### MRI data acquisition

MRI examination was performed by using 3.0 T Philips Health care MRI scanners with 8-channel breast dedicated coils. Patients were maintained in the prone position, and the bilateral breasts naturally and symmetrically fell into the coil. All patients were asked to reduce their respiratory rate to avoid motion artifacts caused by breathing and the heart beating. The contrast agent (Gd-DTPA, 0.1 mmol/kg, Omniscan, GE Healthcare) was injected intravenously at a rate of 2.5 ml/s. Then, 20 ml saline was injected at the same rate to flush out the residual contrast agent. A total of 9 phases were scanned without intervals, and the first phase involved plain scanning. After intravenous injection, continuous noninterval scans were performed in 8 phases. The scan time for each phase was 58 s. The DCE-MRI sequences were acquired using a VIBRANT multiphase sequence as follows: TR/TE: 3.8/1.6 ms, FOV: 300 × 300 mm2, matrix size: 512 × 512, silence thickness: 1.5 mm. The second phase of dynamic contrast enhancement was selected as the object of image segmentation because the peak value of enhancement in the lesion area occurs within 60–120 s after injection of contrast agent [24].

### Patient clinical data recording

All MR images were reviewed by two experienced radiologists (both of them had 6 and 8 years of diagnostic experience, respectively). Clinical data included age, tumor size, histological grade, multifocality, MRI-reported LN status, estrogen receptor (ER), progesterone receptor (PR), human epidermal growth factor receptor 2 (HER2) status and Ki-67 level. Immunohistochemical positive standard occured when tumors contained 10% of immunostained cells defined as ER- or PR-positive. Positivity for HER2 was defined as hematoxylin-eosin (HE) staining at least 3+. KI-67 positivity was defined as at least 14% immunostained cells. ALN metastasis was confirmed by histopathology (macrometastasis or micrometastasis of lymph nodes was defined as positive). MRI-reported LN status for suspicious metastasis was defined as follows: long and short axis lengths exceeding 9 mm and 11 mm, respectively; eccentric cortical thickness over 4 mm; and loss of the fatty hilum [12]. In situations where the two radiologists held different opinions about the details of the clinical characteristics, consensus was reached through consultation.

### Image segmentation and feature extraction

**Fig. 2:**
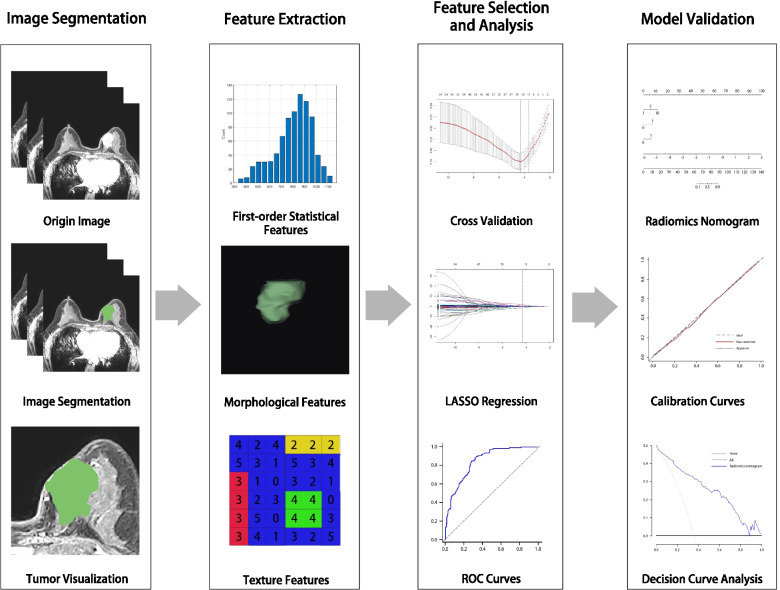
The workflow of this study. This study includes image segmentation, feature extraction, feature selection, feature analysis and model validation. The ROIs of breast cancer was segmented and then 55 quantitative radiomics features were extracted from the segmentation images of individual patients. The least absolute shrinkage and selection operator was used for feature selection and signature construction. The performance of radiomics signature was evaluated by area the receiver operating characteristic curves. Finally, a nomogram was plotted based on the radiomics signature and clinical model, and the predictive performance of the nomogram was validated by the calibration curves and decision curve analysis

**Fig. 3:**
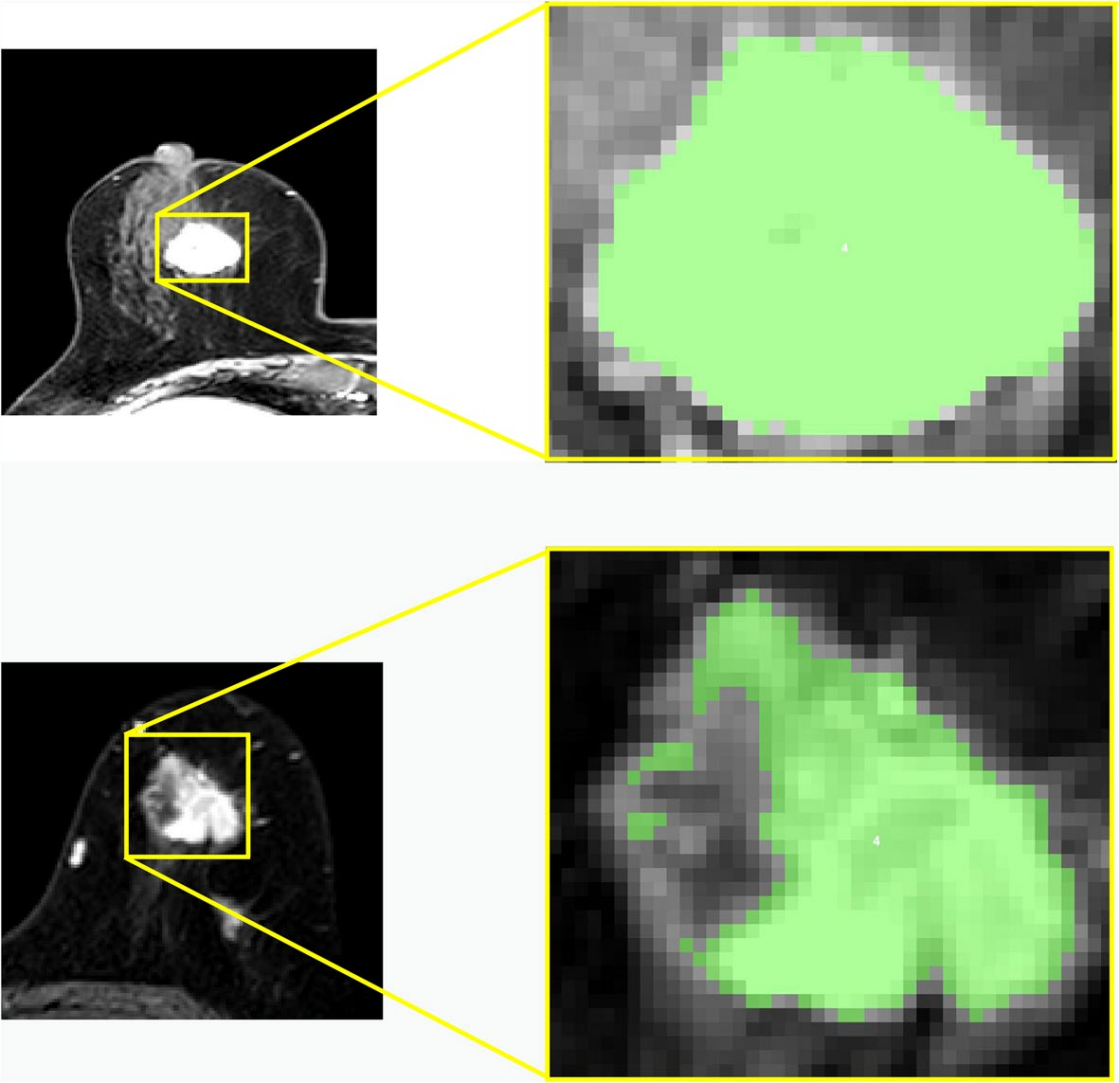
Two pre-processing examples. Left side halves: MR images with ductal carcinoma of breast. Right side halves: Corresponding drawing of partial enlargement under tumor semi-automatic segmentation (indicated in green)

MR images often display intensity nonuniformities due to variations in the magnetic field, which may affect the accuracy of the prediction model. Prior to segmenting MR images, bias field correction was applied to eliminate bias field artifacts and avoid inhomogeneity [25]. The workflow is shown in Fig. 2. MR images were semiautomatically segmented using the open source software MR Radiomics Platform (MRP, http://www.ym.edu.tw/~cflu/MRP_MLinglioma.html). According to Huang et al. [22], radiomics features should be extracted based on the largest breast tumor area rather than the ALN from MR images. The two-dimensional region of interest (ROI) covers the entire tumor area (including enhancing and necrotic regions) as defined by a radiologist with 6 years of experience. The enhancing and hyperintense regions’ pixels in the ROI were first detected by applying a threshold to extract the hyperintense voxels on DCE-MR images, then the same or similar voxels were automatically connected to form target regions by using the regional-growth segmentation algorithm, and the irrelevant voxels of the ROI were eliminated. The ROIs were then manually adjusted and confirmed by a senior radiologist. The diagram of image processing is displayed in Fig. 3.

A total of 55 radiomics features were extracted using the MR Platform, including 14 first-order statistical features, 8 morphological features, 22 Gy-level cooccurrence matrix (GLCM) features and 11 Gy-level run-length matrix (GLRLM) features.

### Statistical analyses

R software (version 3.6.2) was utilized to perform statistical analysis. Continuous variables between the ALN metastasis group and the non-ALN metastasis group were evaluated by Student’s t test or nonparametric Mann–Whitney U test, and categorical variables between the two groups were assessed by Pearson’s chi-squared test where a *p* value of < 0.05 was considered significant.

### Feature selection and signature establishment

The Z score standardization method was used to standardize the corresponding features of each patient to eliminate the unit boundary and quantify and weigh the feature parameters of different units before feature selection. In the training cohort, the least absolute shrinkage and selection operator (LASSO) algorithm was used to screen the optimal features by shrinking the portion of feature coefficients unrelated to ALN metastasis to zero. A radiomics signature was established according to the optimal features and coefficients weighted by the LASSO algorithm, and the discriminant abilities of the radiomics signature in both cohorts were calculated by the area under the curve (AUC).

### Establishment of the clinical model and nomogram

Clinical factors related to ALN metastasis were evaluated by univariable logistic analysis and included age, tumor size, histological grade, multifocality, MRI-reported LN status, ER, PR, HER2 status, Ki-67 level, and radiomics score. Independent clinical factors were screened using the forward selection method in logistic analysis to establish a clinical model.

On the basis of the clinical model, the radiomics signature was added to establish a combined model using multiple logistic regression, and the discriminant ability of the clinical model and combined model was assessed by the AUC. To provide an easy and reliable tool for predicting ALN metastasis, a nomogram based on the combined model was plotted, and then the calibration curve was plotted to demonstrate the probability of predicting ALN metastasis in both cohorts by bootstrapping with 1000 repetitions. The prediction performance of the training and validation cohort nomogram was evaluated using the Hosmer–Lemeshow test. To determine the predictive performance of the radiomics nomogram in clinical use, decision curve analysis (DCA) was performed by quantifying the net benefits at different threshold probabilities in the two cohorts.

## Results

### Patient clinical data

The clinical data are summarized in Table 1. In both cohorts, histological grade, multifocality and MRI-reported LN status had a significant correlation with ALN metastasis (all *p* values < 0.05); patient age, ER status, PR status, HER2 status, and Ki-67 level had no significant correlation with ALN metastasis (all *p* values > 0.05).

**Table 1:** Clinical characters and radiomics score of the training and validation cohort

characteristic	training cohort			Validation cohort		
	Positive for ALN metastasis(*n* = 106)	Negative for ALN metastasis (*n* = 190)	*P* value	Positive for ALN metastasis(*n* = 54)	Negative for ALN metastasis(*n* = 82)	*P* value
Age (mean ± SD)	53.94 ± 9.66	53.84 ± 10.07	0.727	52.35 ± 11.14	53.20 ± 9.17	0.373
Tumor size (mm)	20.37 ± 4.87	19.95 ± 4.67	0.723	20.44 ± 5.88	19.14 ± 4.72	0.500
Histological grade			<0.001*			<0.001*
I	13	56		3	20	
II	64	108		32	53	
III	29	26		19	9	
Multifocality			<0.001*			0.001*
Yes	53	54		29	19	
No	53	136		25	63	
MRI report LN status			<0.001*			0.006*
Yes	74	81		34	32	
No	32	109		20	50	
Estrogen receptor			0.030*			0.734
Positive	84	128		39	57	
Negative	22	62		15	25	
Progesterone receptor			0.930			0.928
Positive	72	130		34	51	
Negative	34	60		20	31	
HER2 status			0.340			0.897
Positive	30	64		12	19	
Negative	76	126		42	63	
Ki-67 status			0.881			0.210
Positive	75	136		41	54	
Negative	31	54		13	28	
Radiomics score	−0.008(−0.55 to 0.36)	−1.642(−2.40 to −0.72)	<0.001*	0.064(−0.47 to 0.52)	−1.613(−2.67 to −0.63)	<0.001*

Note: Data are number of patients; Data in the last line in parentheses are interquartile ranges

*LN* lymph node, *HER2* human epidermal growth factor receptor 2, *SD* standard deviation

^*^highlights the p values that are smaller than 0.05

### Radiomics signature establishment

**Fig. 4:**
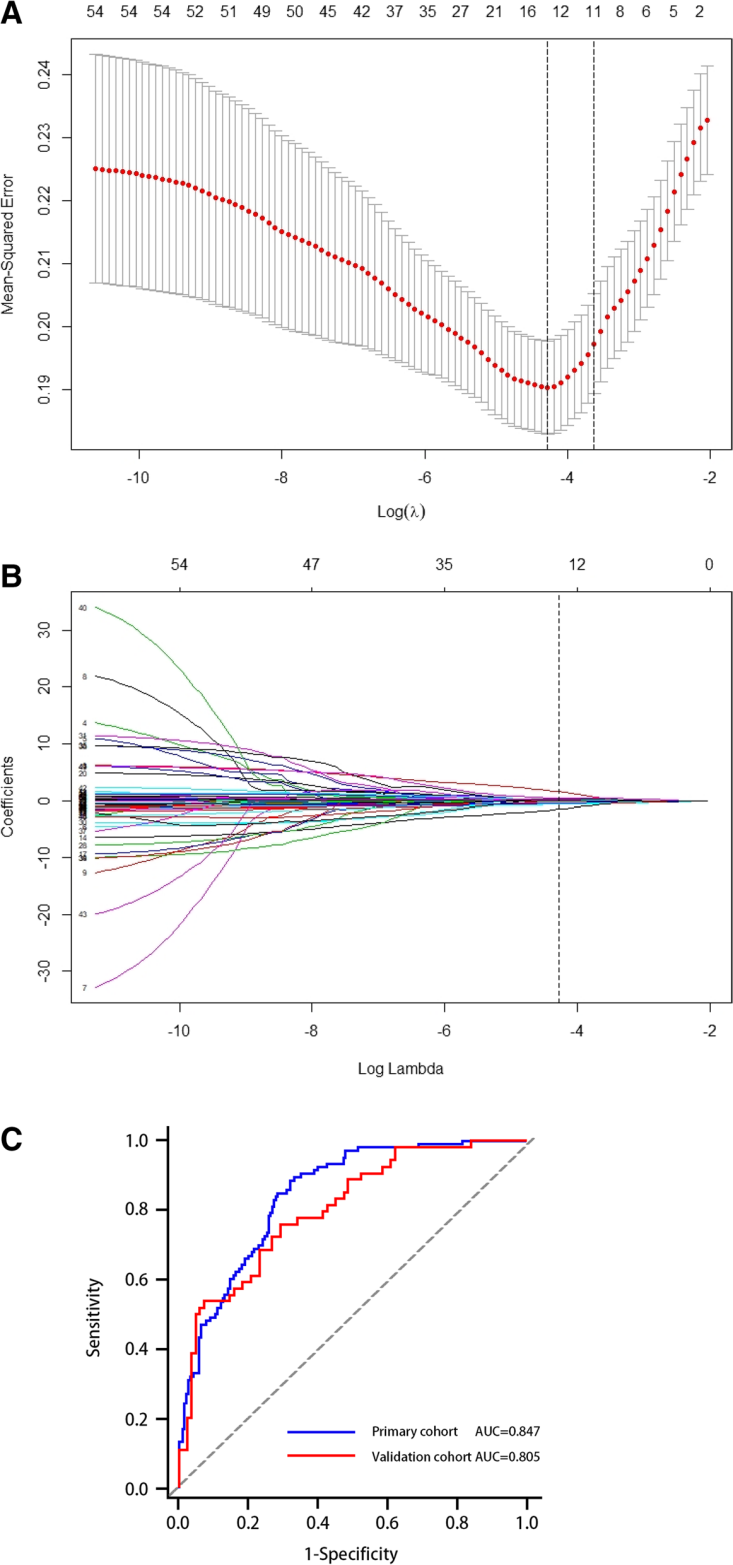
Radiomics feature selection using the LASSO regression algorithm in the primary cohort. **A**. Selection of the parameter (λ) in the LASSO model via 10-fold cross-validation depending on the minimum criteria. The binomial deviance curve versus log (lambda) was plotted, and the left vertical line corresponds to the optimal value of the minimum criterion; the right vertical line corresponds to the optimal value of the 1-SE criteria. The optimal λ value of 0.0127with threshold log (λ) of was − 4.32 was selected. **B**. LASSO coefficient profiles of the 55 features. Vertical line was plotted at the value selected using 10-fold cross-validation, where optimal λ resulted in 14 nonzero coefficients. **C**. The receiver operating characteristic curves (ROC) of the radiomics signature in the training and validation cohorts

In this study, the 55 features extracted from DCE-MR images were regularized by using the LASSO algorithm, and the number of features was reduced from 55 to 14 in the training cohort, including 6 first-order statistical features, 1 morphological feature, 3 GLCM features and 4 GLRLM features (Fig. 4A, B). The details of the features and the corresponding weighted coefficients are presented in Table 2. The radiomics score of each patient was calculated with the 14 optimal features and the corresponding weighted coefficients, and there was a significant difference between the two cohorts (median − 0.008 vs. -1.642; 0.064 vs. -1.613; all *p* values < 0.001) (Table 1). The radiomics signature yielded AUCs of 0.847 [95% confidence interval (CI), 0.801 ~ 0.886] and 0.805 [95% CI, 0.728 ~ 0.868] in the training and validation cohorts, respectively (Fig. 4C).

**Table 2:** List of selected feature parameters for establishing the radiomics signature

Category	Radiomics feature	Coefficient
First order feature (*n* = 6)	Energy	0.273
	Entropy	1.814
	Kurtosis	0.029
	Maximum	0.006
	Mean	−0.630
	Uniformity	−1.713
Morphological feature (*n* = 1)	Spherical disproportion	0.143
GLCM features (*n* = 3)	Correlation	−0.037
	Entropy	−0.421
	Information measure of correlation	−0.614
GLRLM features (*n* = 4)	Run length nonuniformity	0.524
	Short run high gray level emphasis	−0.467
	Short run low gray level emphasis	0.087
	Long run low gray level emphasis	0.136

Note: *GLCM* gray level co-occurrence matrix, *GLRLM* gray level run length matrix

### Establishment and validation of the clinical model and nomogram

**Fig. 5:**
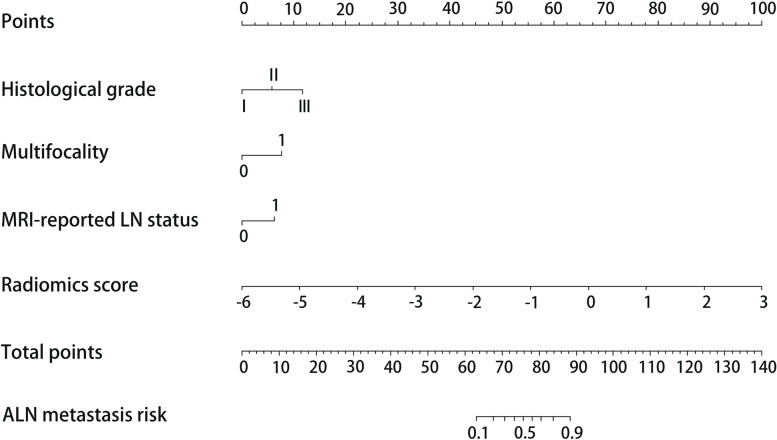
Nomogram was developed with histological grade, multifocality, MRI-reported LN status, and radiomics signature for predicting ALN metastasis in the training cohort

Table 3 shows the results of the multivariable logistic analysis in the training cohort. Independent clinical factors (including histological grade, multifocality and MRI-reported LN status) were used to establish the clinical model, and the combined model was established by incorporating the clinical model and the radiomics signature. To visualize the risk of ALN metastasis for each patient, a nomogram was plotted based on the combined model (Fig. 5).

**Table 3:** Multivariable logistic regression analysis of risk factors for ALN metastasis

Intercept and variable	Combined model in the training cohort		
	Coefficient	Odds ratio (95% CI)	*P* value
Intercept	−2.586	0.073 (0.003–0.100)	3.23 × 10^−5^
Histological grade	1.012	2.751 (1.614–4.877)	3.11 × 10^−4^
Multifocality	1.331	3.783 (1.941–7.601)	1.26 × 10^−4^
MRI report LN status	1.083	2.954 (1.537–5.821)	1.37 × 10^−3^
Radiomics signature	1.927	6.871 (4.121–12.617)	1.25 × 10^−11^

Note: Data in parentheses are 95% confidence intervals. *P* value are displayed as scientific notation. The combined model was established based on these risk factors related to the axillary lymph node metastasis, while the clinical model without radiomics signature

*CI* Confidence interval

**Fig. 6:**
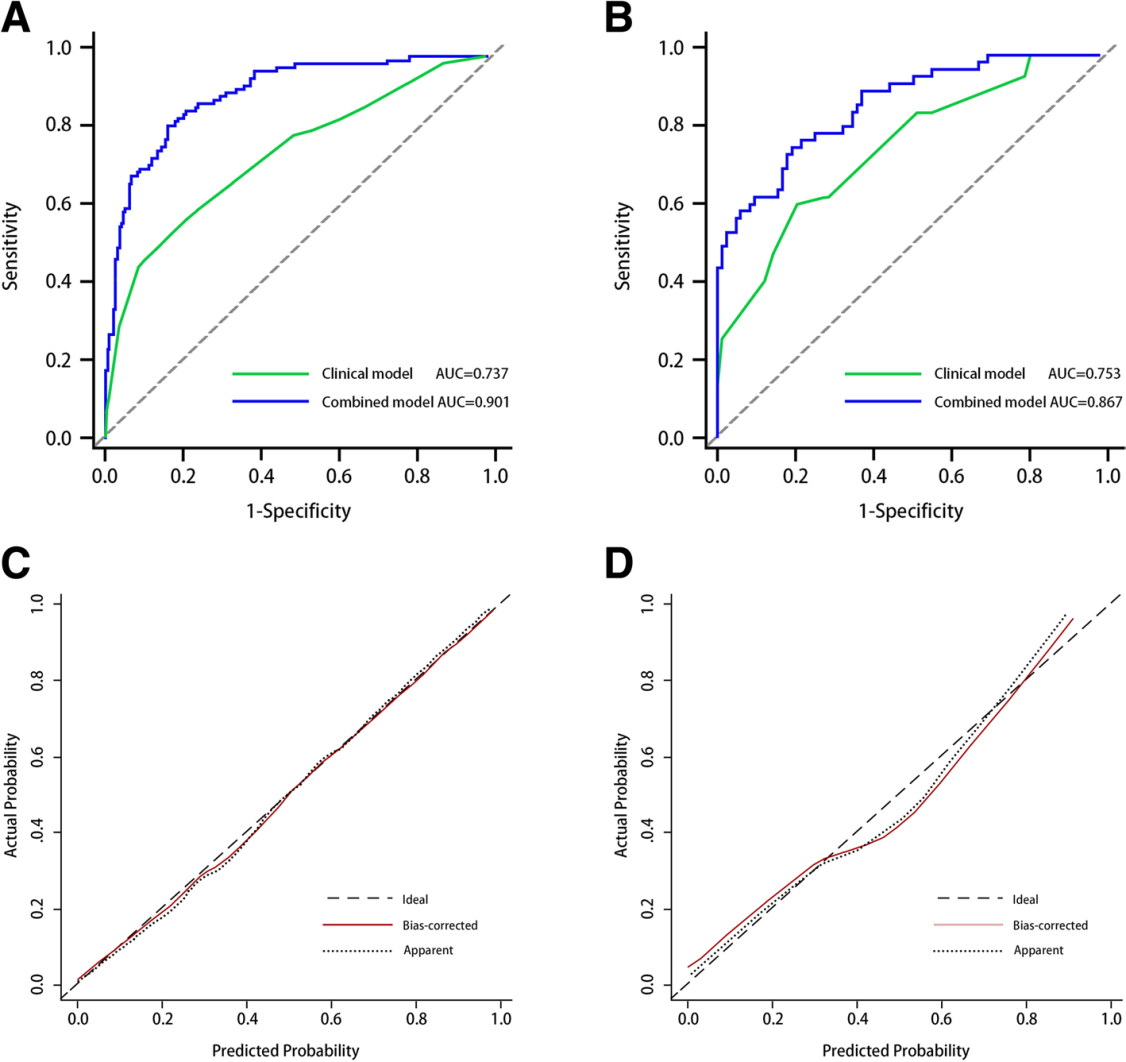
**A** and **B** showed the comparison of receiver operating characteristic curves between the radiomics nomogram and clinical model in the training and validation cohorts, respectively. **C**. Calibration curve of the radiomics nomogram in the training cohort. **D**. validation cohort. The calibration curve was used to estimate the goodness of fit between the actual value (Y-axis) and the predicted value of ALN metastasis (X-axis). The diagonal dashed line represents the predictive performance of the ideal model, the dotted line represents predictive performance of the nomogram, and the red solid line represents the performance of the radiomics nomogram without removed the bias. The closer the two curves are to the diagonal dashed line, the higher the predictive power of the model

**Fig. 7:**
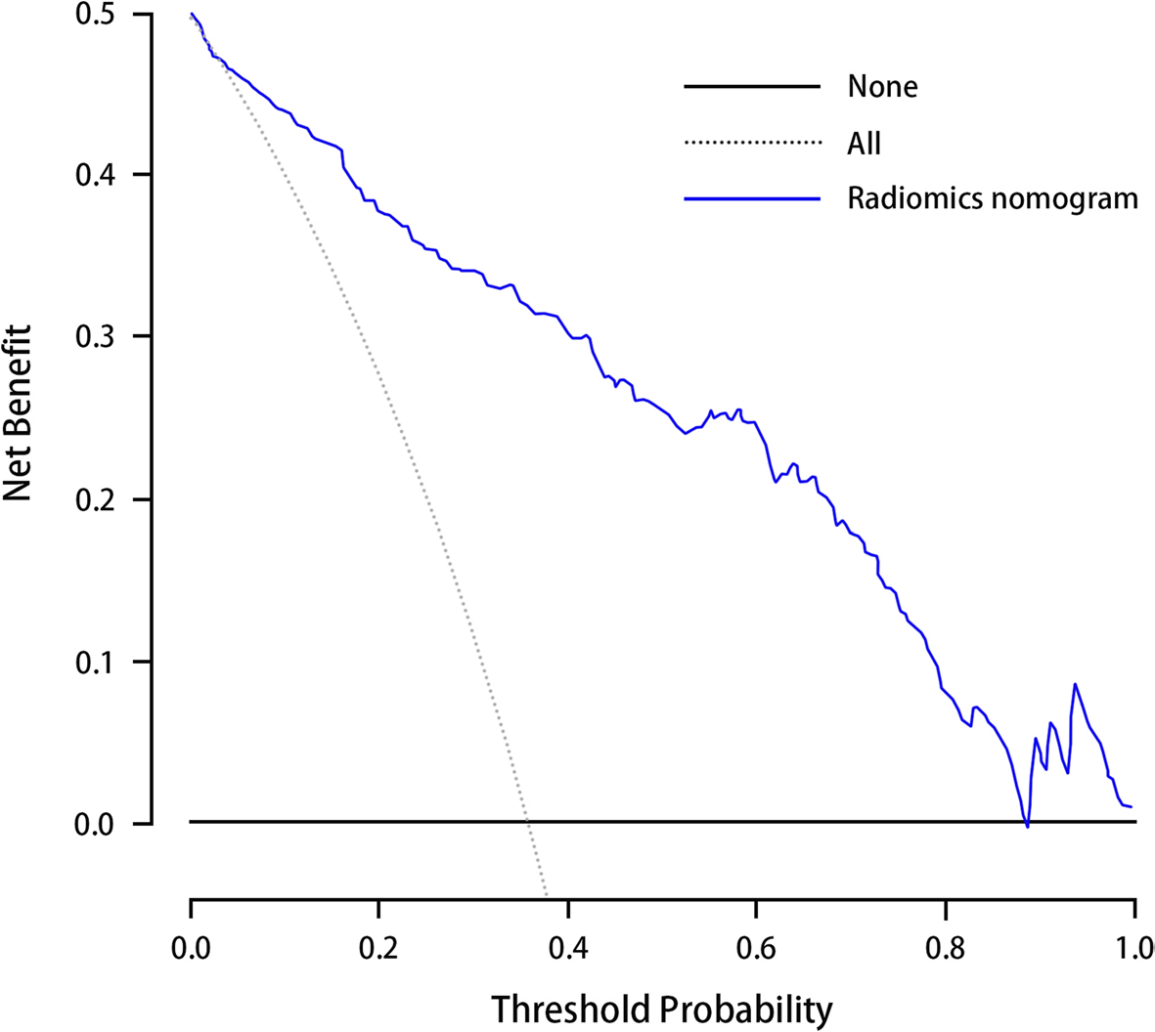
Decision curve analysis for radiomics nomogram in the training cohort. The x-axis indicates the threshold probability and y-axis measures the net benefit. The blue line represents the radiomics nomogram. The gray line represents the assumption that all patients have LN metastases. The black line represents the assumption that no patients have LN metastases. The decision curve showed that when the threshold probability is between 0.04 to 0.88, more benefit was added from the radiomics nomogram than either the treat-all-patients scheme or the treat-none scheme

As shown in Fig. 6A and B, the clinical model showed moderate predictive performance, with AUCs of 0.723 [95% CI, 0.682 ~ 0.785] and 0.738 [95% CI, 0.656 ~ 0.810] in the training and validation cohorts, respectively. The AUC of the clinical model validation cohort was higher than that of the training cohort. The specificity of the clinical model was good, registering as high as 82.63 and 79.27% in the training and validation cohorts, respectively, but the sensitivity was poor, measuring only 53.77 and 61.11% in the training and validation cohorts, respectively. The combined model demonstrated better discrimination than the clinical model and radiomics signature alone, which yielded AUCs of 0.907 [95% CI, 0.868 ~ 0.937] and 0.874 [95% CI, 0.807 ~ 0.925] in the training and validation cohorts, respectively (Table 4). The predictive value of the calibration curves in the two cohorts had good consistency with the actual results (Fig. 6C, D). The results of the Hosmer–Lemeshow test showed a nonsignificant difference (*p* values of 0.152 and 0.246 in both cohorts**,** respectively). DCA reflected the clinical utility of evaluating the performance of the nomogram. Figure 7 shows the DCA for evaluating ALN metastasis based on the radiomics nomogram. When the threshold probability was in the range of 0.04–0.88, the maximum net benefit could be obtained by using the radiomics nomogram to predict ALN metastasis.

**Table 4:** Prediction performance in the training and validation cohorts

	AUC	Sensitivity	Specificity	Threshold
Radiomics signature				
Training cohort	0.847 (0.801, 0.886)	88.68%	67.89%	0.566
Validation cohort	0.805 (0.728, 0.868)	75.93%	70.73%	0.467
Clinical model				
Training cohort	0.737 (0.683, 0.787)	53.77%	82.63%	0.364
Validation cohort	0.753 (0.672, 0.823)	61.11%	79.27%	0.404
Combined model				
Training cohort	0.907 (0.861, 0.932)	82.08%	83.65%	0.657
Validation cohort	0.867 (0.798, 0.919)	75.93%	80.49%	0.564

Note: Data in parentheses are 95% confidence intervals

*AUC* indicates area under the curve

## Discussion

In this study, we developed a radiomics nomogram based on primary tumor characteristics for predicting ALN metastasis in breast cancer. This multivariable model is composed of features extracted from DCE-MR images and clinical factors, and it displayed excellent ability, with an AUC of 0.907 in the training cohort and 0.874 in the validation cohort. The application of radiomics features extracted from DCE-MR images provides a new method for quantifying intratumoral heterogeneity.

At present, with the continuous exploration of radiomics, an increasing number of studies have shown that it can be used as a powerful noninvasive method to increase detection, diagnosis, and prediction [26, 27]. Thus, in this study, we developed and validated a radiomics signature based on DCE-MRI radiomics features for predicting ALN metastasis in breast cancer. A total of 14 optimal features were finally screened by the LASSO algorithm and used to construct the radiomics signature. The discriminative ability of the radiomics signature was impressive, with AUCs of 0.847 and 0.805 in both cohorts. Recently, Han et al. [28] developed a nomogram based on the radiomics features of the first enhancement phase of DCE-MRI to preoperatively evaluate ALN status, with AUCs of 0.76 and 0.78 in the primary and validation cohorts, respectively. In the present work, we used the second phases of tumor enhancement and achieved higher predictive performance. Compared with the study of Han et al. [28], the ROIs in the second enhancement phases showed the lesion boundaries more clearly. Routine T2-weighted imaging (T2WI) and diffusion-weighted MRI (DWI) sequences were not included in the present work. In fact, the border of the tumor is not clear on the T2WI and DWI images, and it is difficult to completely segment the tumor lesion. In the study by Dong [29], radiomics features from the T2WI and DWI images were used to predict SLN metastasis and obtained relatively low AUC values of 0.770 and 0.787. Furthermore, other studies have reported that the strongest enhancement phase in DCE-MRI can better reflect the heterogeneity and aggressiveness of the tumor [30].

Among the 14 radiomics features, the 4 first-order features were consistent with previous studies. Wu et al. [23] demonstrated that first-order features had reliable performance in predicting the LN metastasis of bladder cancer, which could help patients who are LN negative avoid unnecessary pelvic LN dissection and neoadjuvant chemotherapy. GLCM and GLRLM features are widely used texture parameters in the field of radiomics and machine learning [31], which can reveal minute changes in the tumor histological anatomy. In this study, Entropy, Information Measure of Correlation and Short Run Emphasis are all in line with previously reported features related to ALN metastasis extracted from the first enhancement phase of DCE-MRI and could reflect the degree of tumor heterogeneity and invasiveness based on the radiomics score [28, 32]. However, we use different enhancement phases and feature extraction methods. Previous work suggested that GLCM parameters extracted from MR images are correlated with LN metastasis and can better display the heterogeneity and complexity of the intratumor microenvironment [33]. Most importantly, GLCM and GLRLM features could be regarded as biomarkers to stratify patients with breast cancer more precisely [32].

In this study, we determined some clinical factors associated with ALN metastasis, including histological grade, multifocality and MRI-reported LN status. As expected, these ALN metastasis-associated predictive factors were quite similar to those identified in previous studies [34–36]. Therefore, to provide an easy and feasible tool for clinicians, a nomogram that incorporates a clinical model and radiomics signature was established to improve the diagnostic efficiency and visualize the risk score of individual ALN metastasis prediction. We were especially encouraged by the good discriminability of the nomogram (AUCs of 0.907 and 0.874 in both cohorts), and the calibration curves demonstrated good consistency between the predicted value and the actual outcome. Our study confirmed that SLNB can be omitted or ALND can be performed directly without lymph node biopsy for patients with high-risk grade. To date, four similar studies have developed predictive models for predicting ALN metastasis based on DCE-MRI. The data of our radiomics models in our study were all from the same hospitals in China, repeating the same protocol for all patients, which increases the reproducibility of feature extraction in the research process. In addition, the region-growing segmentation algorithm was performed on the ROIs to avoid the influence of irrelevant voxels in the calculation of feature parameters.

Recently, Yang et al. [37] developed a radiomics nomogram based on mammogram features to preoperatively evaluate ALN status, with AUCs of 0.779 and 0.809 in the primary and validation cohorts, respectively. Furthermore, Yu [38] and colleagues applied radiomic features extracted from ultrasound images to predict ALN metastasis in breast cancer, and the prediction performance was not satisfactory (AUC = 0.78). The results of our study are superior to those of previous studies since radiomics feature extraction was based on DCE-MR images. It is also possible that DCE-MRI can provide richer intratumoral hemodynamic features for radiomics analysis. In addition, the AUC value in our study was slightly higher than that of the study of Cui et al. [39], and the AUC value of the SVM classifier was 0.861 in predicting ALN in breast cancer using the radiomics features of DCE-MR images. As with our study, Santucci et al. [40] also used the second contrast enhancement phase to evaluate the ALN status of breast cancer, and the AUC of the random forest prediction model reached 0.856. This may have some association with the number of patients and classifiers. The small sample sizes of 102 patients may limit the clinical applicability of the model. However, our prediction efficiencies were slightly lower than those in the study of Yu et al. [41], and their AUC values were 0.92 in the primary cohort and 0.90 in the validation cohort in predicting ALN metastasis of breast cancer using the radiomics features of DCE-MRI. This may be related to two main reasons. One reason is that the prediction performance of the clinical model is higher than ours (AUC = 0.77). Another reason is the number of patients enrolled in our study. We only have a total of 432 patient data points, and the dataset in their study involved more than 1000 patients from 4 medical institutions.

The study has some limitations. First, this is a retrospective study with a limited sample size, and the subjects assessed for the construction and validation of our predictive model were all from the same hospital in China. Although the radiomics nomogram shows comparable performance, a larger sample size from multiple centers needs to be used to verify the model and provide reliable evidence for clinical application. Second, the ROI outline of breast cancer lesions was delineated by the semiautomatic segmentation method. We tried to avoid hemorrhagic or edematous areas by adjusting the threshold, but discernment of the lesion’s exact outline was greatly influenced by radiologist’s experience. Furthermore, we selected the primary tumor area instead of the LN area as the ROI in our study for feature extraction since it was difficult to match the biopsied LNs with those on the MR images. The results of our study confirmed that the changes in MR image features in the primary tumor area of breast cancer are related to ALN metastasis, and we expect that background parenchymal enhancement and peritumoral region features may further improve the prediction performance of the model [42, 43].

## Conclusion

In conclusion, the radiomics features extracted from the primary tumor area of DCE-MR images can be used as potential biomarkers to predict ALN metastasis. In this study, we exploited a nomogram by incorporating a radiomics signature and a clinical model, which can provide valuable evidence to support clinical operation and treatment decision-making. This study is a step toward precision medicine and personalized treatment for breast cancer patients. Further studies based on radiomics are expected to make greater contributions to the diagnosis, staging and treatment of breast cancer.

## Supplementary Information

**Additional file 1.**

**Additional file 2.**

**Additional file 3.**

**Additional file 4.**

**Additional file 5.**

**Additional file 6.**

## Data Availability

The datasets used and/or analyzed during the current study available from the corresponding author on reasonable request.

## Abbreviation

ALN: Axillary lymph node
SLN: Sentinel lymph node biopsy
LASSO: Least absolute shrinkage and selection operator
ROI: Regions of interest
ROC: Receiver operating characteristic curve
AUC: Area under the receiver operating characteristic curve
GLCM: Grey-level co-occurrence matrix
GLRLM: Gray level run length matrix
