# Supplementary material for: Dynamic contrast-enhanced MRI radiomics nomogram for predicting axillary lymph node metastasis in breast cancer
Source: Cancer Imaging. 2022 Apr 4;22:17. doi: 10.1186/s40644-022-00450-w (PMC8981871; doi:10.1186/s40644-022-00450-w)
Supplement: Supplementary file 3 — Additional file 3. [file 40644_2022_450_MOESM3_ESM.docx]

**research process**

**The first step: image preprocessing**

Software: python pytorch

Code

import SimpleITK as sitk

import warnings

import glob

import numpy as np

import cv2

import numpy

import dicom

from matplotlib import pyplot as plt

imagePath = '/ Users/desk/data/breastcancer/YUAN LI PING.dcm '

input_image = sitk.readImage(imagePath)

mask_image = sitk.OtsuThreshold(input_image, 0, 1, 200)

input_image = sitk.Cast(input_image, sitk.sitkFloat32)

corrector = N4BiasFieldCorrectionImageFilter()

output_image = corrector.Execute(input_image, input_image)

output_image = sitk.Cast(output_image, sitk.sitkFloat16)

sitk.writeImage(output_image, '/Users/desk/data/breastcancer/YUAN LI PING.N4.dcm/'

**The second step, image processing**

Software: MATLAB 2018b MR Radiomics Platform

MR Radiomics Platform (MRP, <http://www.ym.edu.tw/~cflu/MRP_MLinglioma.html>).

Enter the software


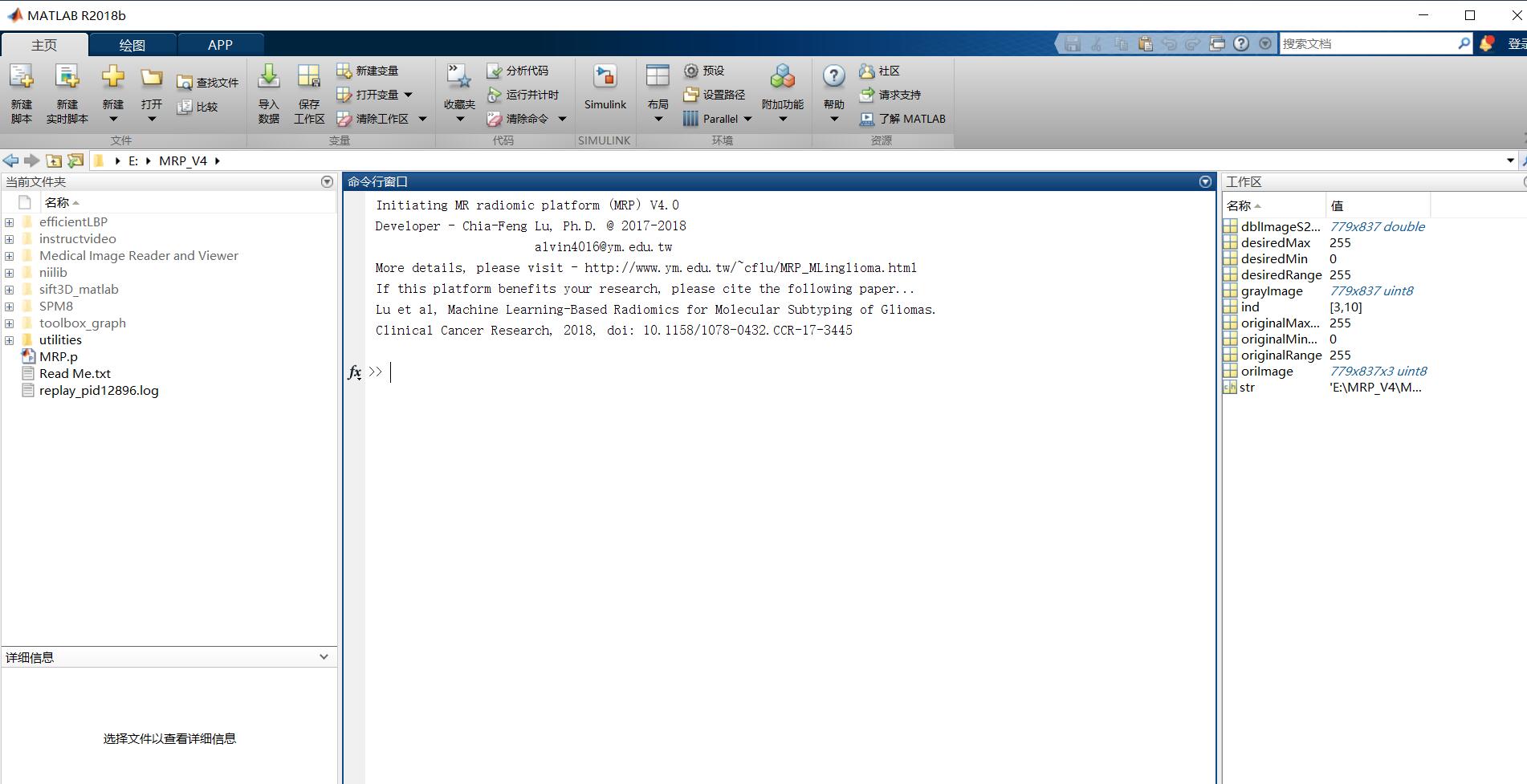


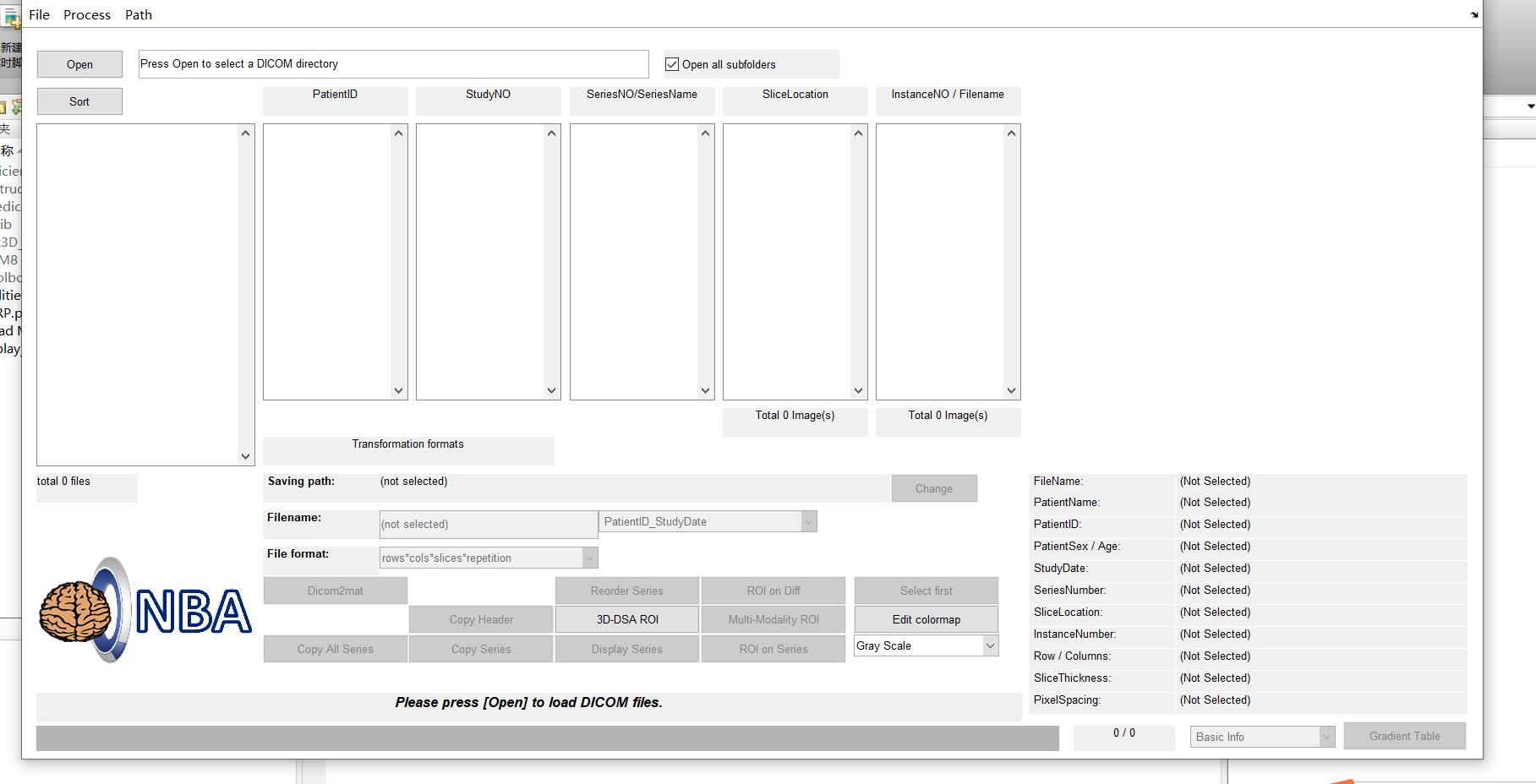


Read image


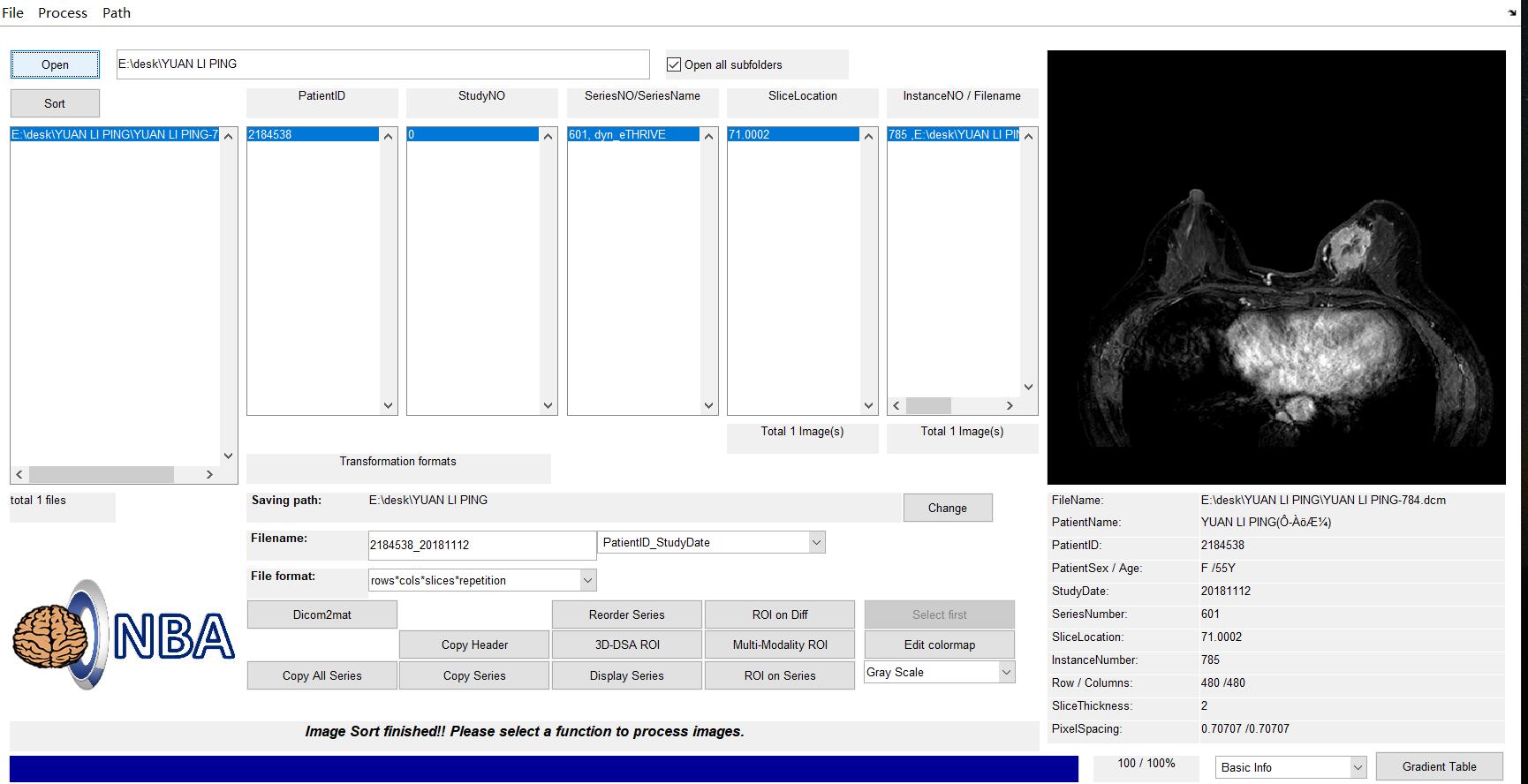


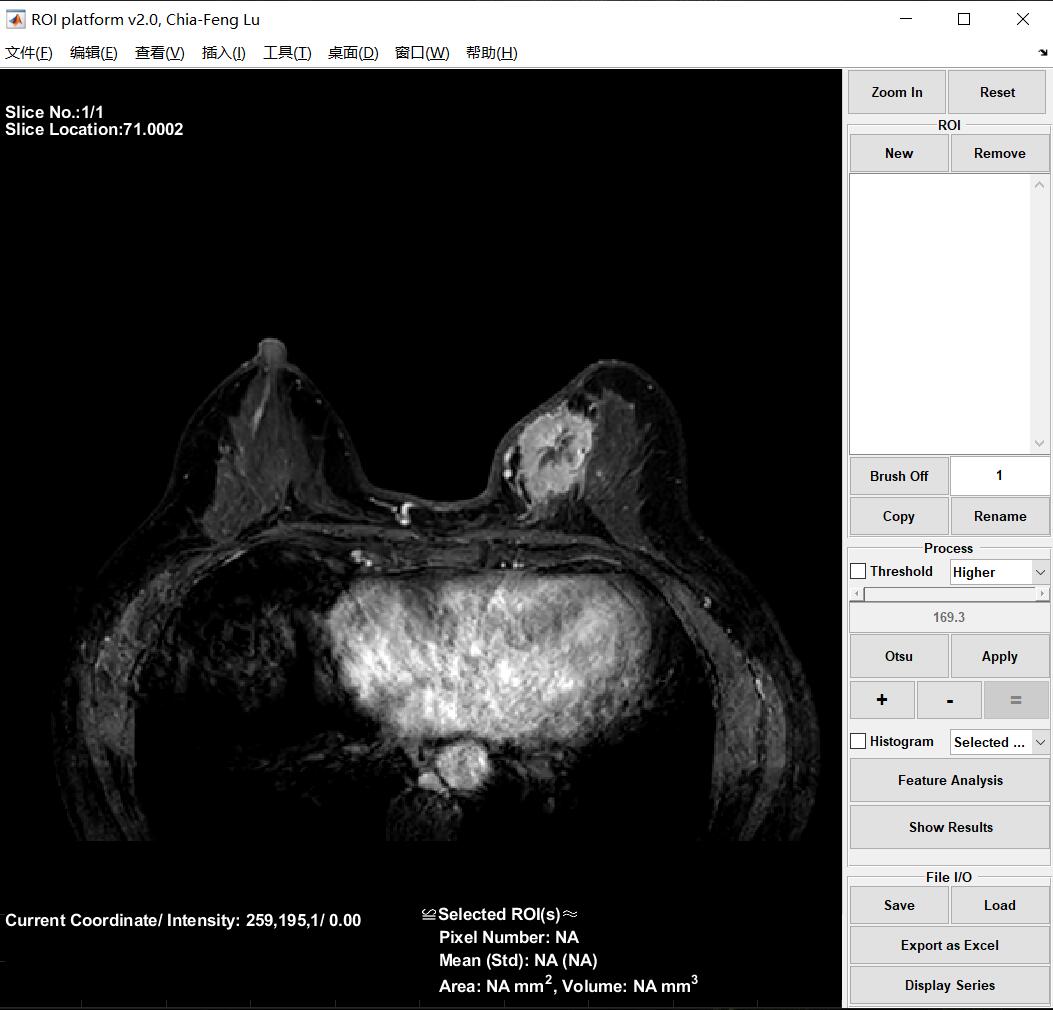
Draw ROI


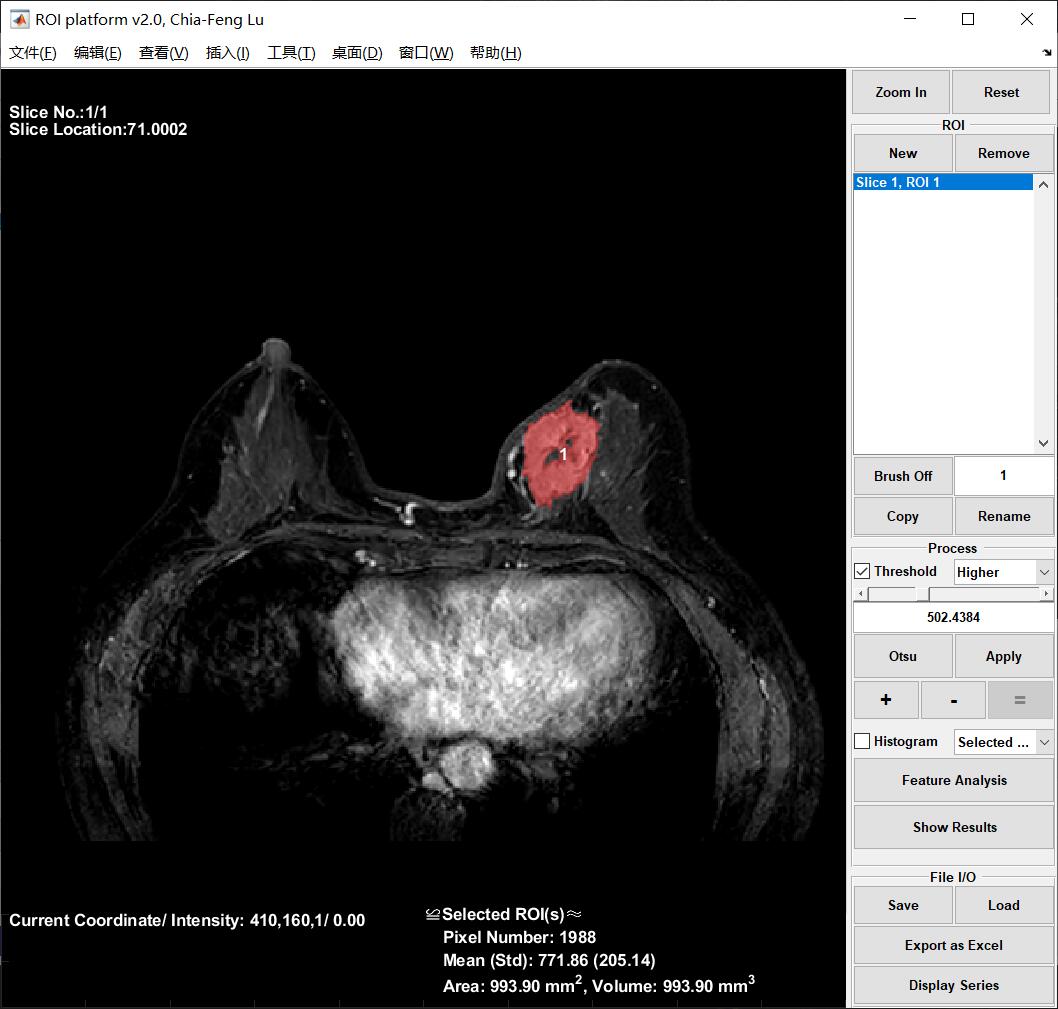


Calculate radiomics parameters


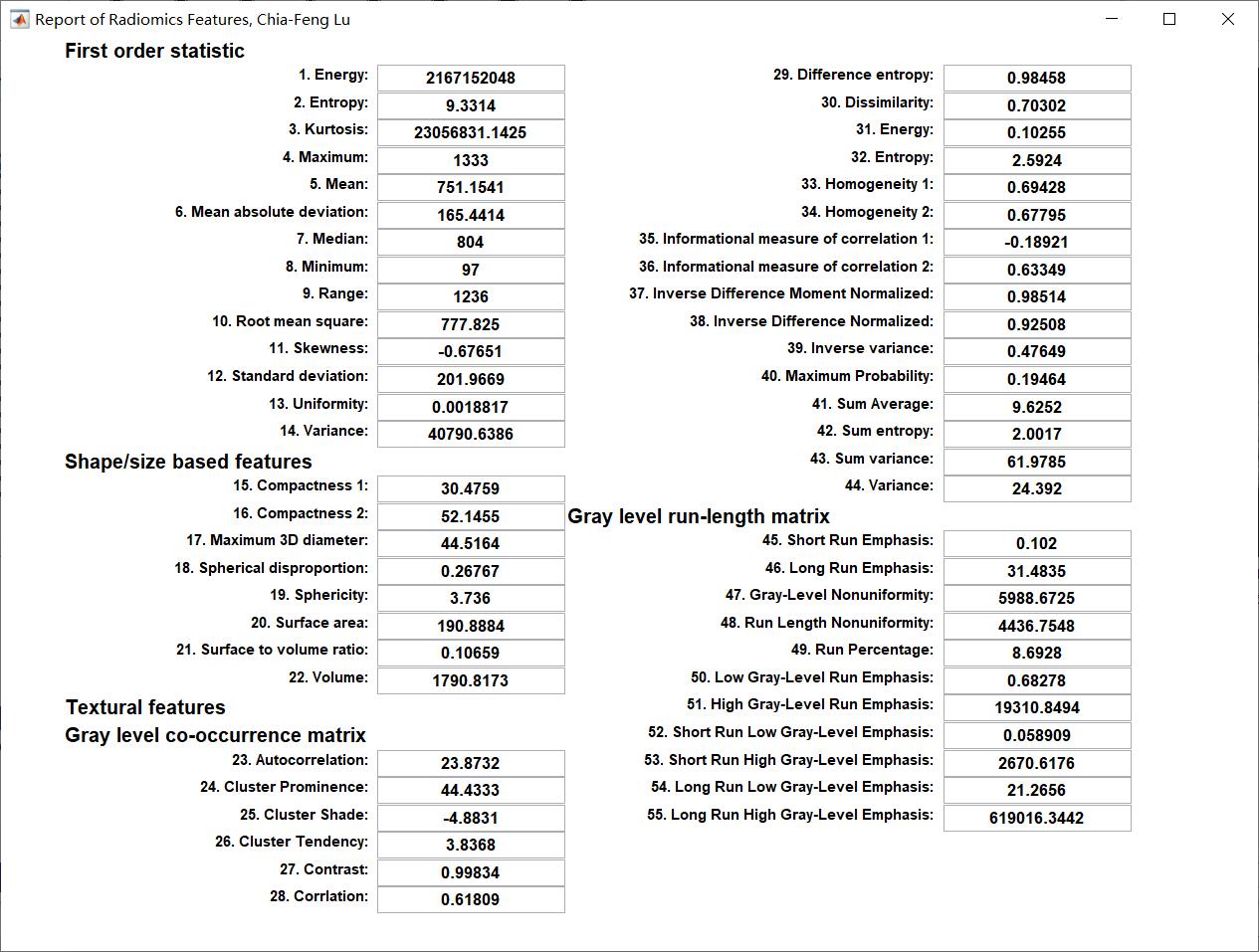


**feature screening**

Software R 3.6.2 Statistics package glmnet

code

data1 <- read.csv('E:\\desk\\training.csv')

x <- model.matrix(CLASS~.,data1)

x <- x[,-1]

y <- data1$CLASS

fit <- glmnet(x,y,alpha = 1,family = 'binomial')

plot(fit,xvar = "lambda",label = TRUE)

print(fit)

cv.fit <- cv.glmnet(x,y,alpha=1)

plot(cv.fit)

abline(v=log(c(cv.fit$lambda.min,cv.fit$lambda.1se)),lty=2)

plot(fit,xvar = "lambda",label = TRUE)

abline(v=log(c(cv.fit$lambda.min)),lty=1)

cv.fit$lambda.min

Coefficients <- coef(fit, s = cv.fit$lambda.min)

Active.Index <- which(Coefficients != 0)

Active.Coefficients <- Coefficients[Active.Index]

Active.Index

Active.Coefficients

row.names(Coefficients)[Active.Index]

predict(fit,s = cv.fit$lambda.min,type = "coefficients")


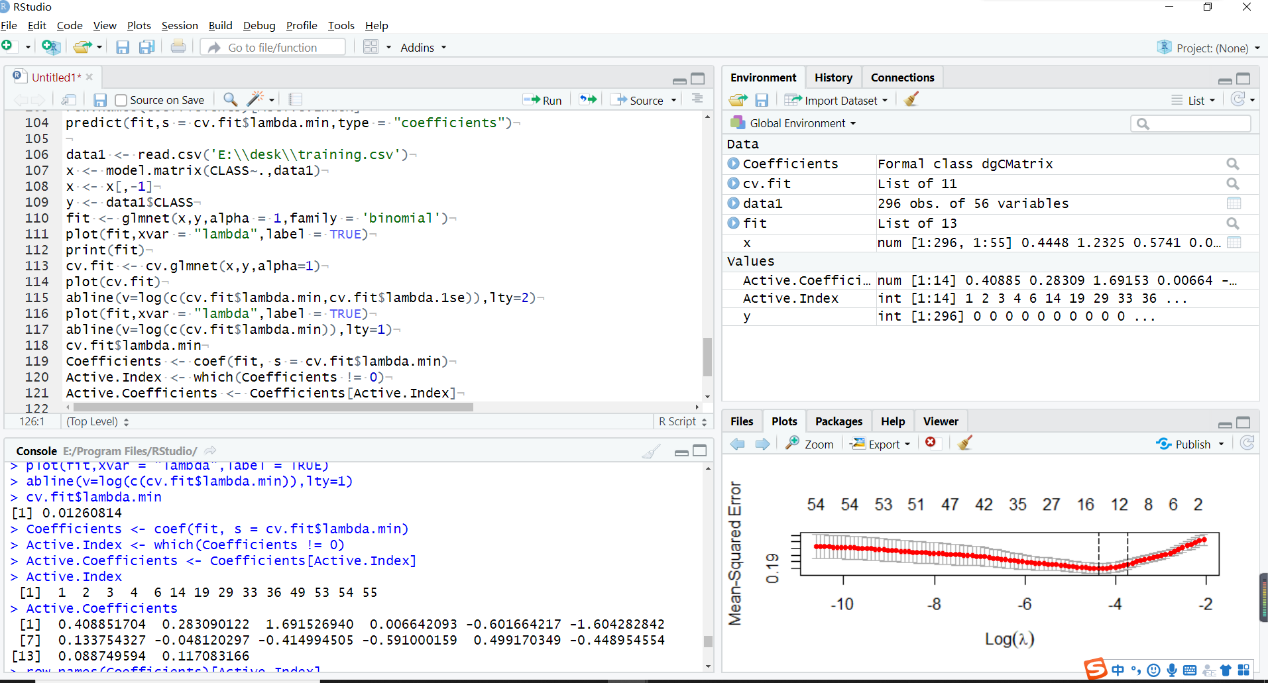
**import results**


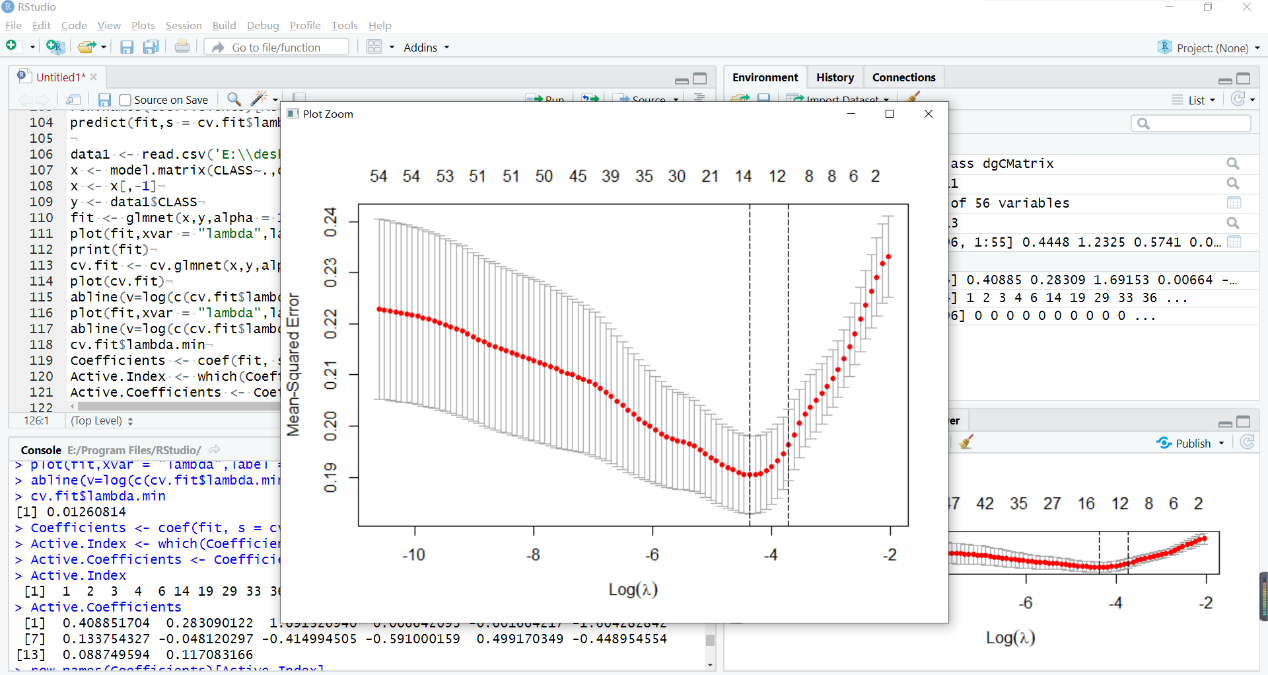


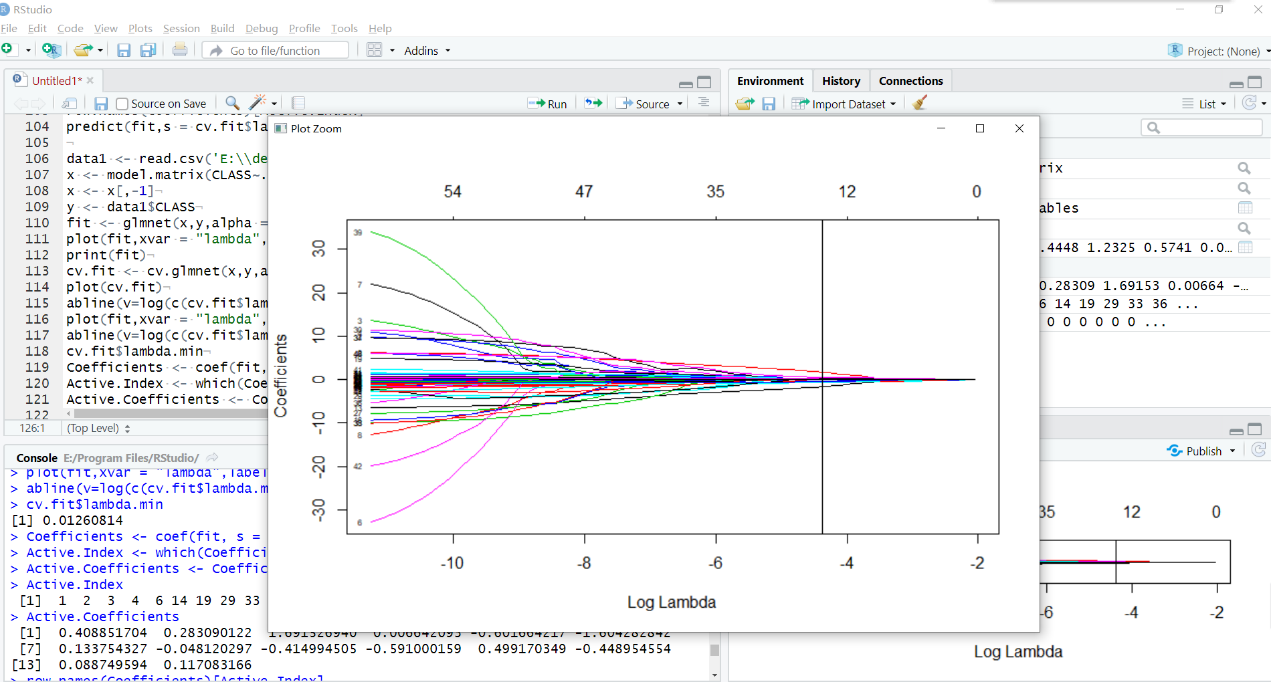


| data1 <- read.csv('E:\\desk\\training.csv')  > x <- model.matrix(CLASS~.,data1)  > x <- x[,-1]  > y <- data1$CLASS  > fit <- glmnet(x,y,alpha = 1,family = 'binomial')  > plot(fit,xvar = "lambda",label = TRUE)  > print(fit)  Call: glmnet(x = x, y = y, family = "binomial", alpha = 1)  Df %Dev Lambda  1 0 0.00000 0.129000  2 2 0.01067 0.117600  3 2 0.02378 0.107100  4 4 0.03742 0.097620  5 4 0.05096 0.088950  6 5 0.06653 0.081050  7 5 0.08037 0.073850  8 6 0.09269 0.067290  9 6 0.10340 0.061310  10 6 0.11280 0.055860  11 8 0.12150 0.050900  12 8 0.13230 0.046380  13 8 0.14190 0.042260  14 8 0.15040 0.038500  15 9 0.15810 0.035080  16 10 0.17180 0.031970  17 10 0.18470 0.029130  18 11 0.19660 0.026540  19 11 0.20730 0.024180  20 11 0.21680 0.022030  21 12 0.22540 0.020080  22 12 0.23330 0.018290  23 12 0.24030 0.016670  24 12 0.24650 0.015190  25 12 0.25200 0.013840  26 13 0.25690 0.012610  27 14 0.26170 0.011490  28 16 0.26790 0.010470  29 16 0.27360 0.009538  30 17 0.27900 0.008690  31 17 0.28480 0.007918  32 18 0.29140 0.007215  33 19 0.29800 0.006574  34 20 0.30530 0.005990  35 21 0.31180 0.005458  36 24 0.31890 0.004973  37 26 0.32560 0.004531  38 28 0.33250 0.004129  39 30 0.33960 0.003762  40 32 0.34650 0.003428  41 33 0.35270 0.003123  42 34 0.35840 0.002846  43 35 0.36370 0.002593  44 35 0.36840 0.002363  45 36 0.37260 0.002153  46 38 0.37660 0.001961  47 39 0.38070 0.001787  48 40 0.38490 0.001628  49 40 0.38890 0.001484  50 41 0.39260 0.001352  51 43 0.39630 0.001232  52 44 0.39950 0.001122  53 43 0.40300 0.001023  54 43 0.40600 0.000932  55 43 0.40850 0.000849  56 42 0.41070 0.000774  57 42 0.41240 0.000705  58 42 0.41380 0.000642  59 43 0.41510 0.000585  60 45 0.41660 0.000533  61 47 0.41880 0.000486  62 48 0.42120 0.000443  63 48 0.42330 0.000403  64 47 0.42520 0.000368  65 47 0.42690 0.000335  66 47 0.42840 0.000305  67 49 0.42970 0.000278  68 50 0.43080 0.000253  69 50 0.43210 0.000231  70 49 0.43420 0.000210  71 49 0.43440 0.000192  72 48 0.43490 0.000175  73 50 0.43630 0.000159  74 49 0.43700 0.000145  75 50 0.43800 0.000132  76 50 0.44000 0.000120  77 50 0.44160 0.000110  78 51 0.44290 0.000100  79 51 0.44400 0.000091  80 52 0.44500 0.000083  81 53 0.44600 0.000076  82 52 0.44680 0.000069  83 53 0.44760 0.000063  84 54 0.44820 0.000057  85 54 0.44880 0.000052  86 54 0.44940 0.000047  87 54 0.45000 0.000043  88 54 0.45040 0.000039  89 54 0.45090 0.000036  90 54 0.45130 0.000033  91 54 0.45160 0.000030  92 54 0.45190 0.000027  93 54 0.45220 0.000025  94 54 0.45240 0.000023  95 54 0.45270 0.000021  96 54 0.45290 0.000019  97 54 0.45310 0.000017  98 55 0.45320 0.000016  99 55 0.45340 0.000014  100 55 0.45350 0.000013  > cv.fit <- cv.glmnet(x,y,alpha=1)  > plot(cv.fit)  > abline(v=log(c(cv.fit$lambda.min,cv.fit$lambda.1se)),lty=2)  > abline(v=log(c(cv.fit$lambda.min,cv.fit$lambda.1se)),lty=2)  > plot(fit,xvar = "lambda",label = TRUE)  > abline(v=log(c(cv.fit$lambda.min)),lty=1)  > cv.fit$lambda.min  [1] 0.01260814  > Coefficients <- coef(fit, s = cv.fit$lambda.min)  > Active.Index <- which(Coefficients != 0)  > Active.Coefficients <- Coefficients[Active.Index]  > Active.Index  [1] 1 2 3 4 6 14 19 29 33 36 49 53 54 55  > Active.Coefficients  [1] 0.408851704 0.283090122 1.691526940 0.006642093 -0.601664217 -1.604282842  [7] 0.133754327 -0.048120297 -0.414994505 -0.591000159 0.499170349 -0.448954554  [13] 0.088749594 0.117083166  > row.names(Coefficients)[Active.Index]  [1] "(Intercept)"  [2] "X1..Energy"  [3] "X2..Entropy"  [4] "X3..Kurtosis"  [5] "X5..Mean"  [6] "X13..Uniformity"  [7] "X18..Spherical.disproportion"  [8] "X28..Corrlation"  [9] "X32..Entropy"  [10] "X35..Informational.measure.of.correlation.1..IMC1."  [11] "X48..Run.Length.Nonuniformity..RLN."  [12] "X52..Short.Run.Low.Gray.Level.Emphasis..SRLGLE."  [13] "X53..Short.Run.High.Gray.Level.Emphasis..SRHGLE."  [14] "X54..Long.Run.Low.Gray.Level.Emphasis..LRLGLE."  > predict(fit,s = cv.fit$lambda.min,type = "coefficients")  56 x 1 sparse Matrix of class "dgCMatrix"  1  (Intercept) 0.408851704  X1..Energy 0.283090122  X2..Entropy 1.691526940  X3..Kurtosis 0.006642093  X4..Maximum .  X5..Mean -0.601664217  X6..Mean.absolute.deviation .  X7..Median .  X8.Minimum .  X9..Range .  X10..Root.mean.square .  X11..Skewness .  X12..Standard.deviation .  X13..Uniformity -1.604282842  X14..Variance .  X15..Compactness.1 .  X16..Compactness.2 .  X17..Maximum.3D.diameter .  X18..Spherical.disproportion 0.133754327  X19..Sphericity .  X20..Surface.area .  X21..Surface.to.volume.ratio .  X22..Volume .  X23..Autocorrelation .  X24..Cluster.Prominence .  X25..Cluster.Shade .  X26..Cluster.Tendency .  X27..Contrast .  X28..Corrlation -0.048120297  X29..Difference.entropy .  X30..Dissimilarity .  X31..Energy .  X32..Entropy -0.414994505  X33..Homogeneity.1 .  X34..Homogeneity.2 .  X35..Informational.measure.of.correlation.1..IMC1. -0.591000159  X36..Informational.measure.of.correlation.2..IMC2. .  X37..Inverse.Difference.Moment.Normalized..IDMN. .  X38..Inverse.Difference.Normalized..IDN. .  X39..Inverse.variance .  X40..maximum.Probability .  X41..Sum.Average .  X42..Sum.entropy .  X43..Sum.variance .  X44..Variance .  X45..Short.Run.Emphasis..SRE. .  X46..Long.Run.Emphasis..LRE. .  X47..Long.Run.Emphasis..LRE. .  X48..Run.Length.Nonuniformity..RLN. 0.499170349  X49..Run.Percentage..RP. .  X50..Low.Grag.Level.Run.Emphsis..LGLRE. .  X51..High.Grag.Level.Run.Emphsis..HGLRE. .  X52..Short.Run.Low.Gray.Level.Emphasis..SRLGLE. -0.448954554  X53..Short.Run.High.Gray.Level.Emphasis..SRHGLE. 0.088749594  X54..Long.Run.Low.Gray.Level.Emphasis..LRLGLE. 0.117083166  X55..Long.Run.High.Gray.Level.Emphasis..LRHGLE. . |
| --- |
| **Radiomics signature** |
| \|  \| \| --- \| |
| 0.408851704＋X1..Energy ×0.283090122 ＋ X2..Entropy ×1.691526940 ＋ X3..Kurtosis× 0.006642093 ＋Mean ×-0.601664217＋Uniformity× -1.604282842＋Spherical.disproportion× 0.133754327 ＋Corrlation× -0.048120297 ＋Entropy× -0.414994505＋Informational.measure.of.correlation.1..IMC1× -0.591000159 ＋ Run.Length.Nonuniformity..RLN× 0.499170349＋Short.Run.Low.Gray.Level.Emphasis..SRLGLE× -0.448954554＋Short.Run.High.Gray.Level.Emphasis..SRHGLE× 0.088749594 ＋ Long.Run.Low.Gray.Level.Emphasis..LRLGLE ×0.117083166 |


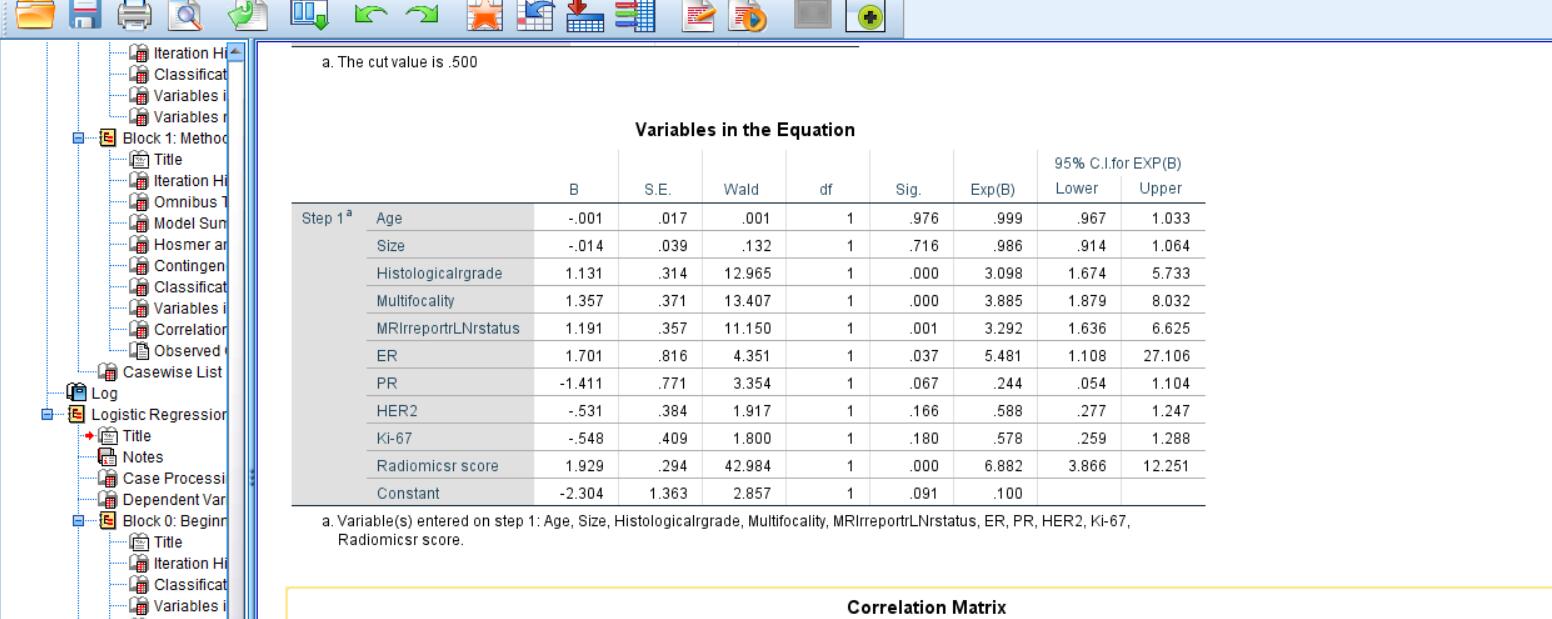
**The statistical analysis of clinical data uses SPSS software**


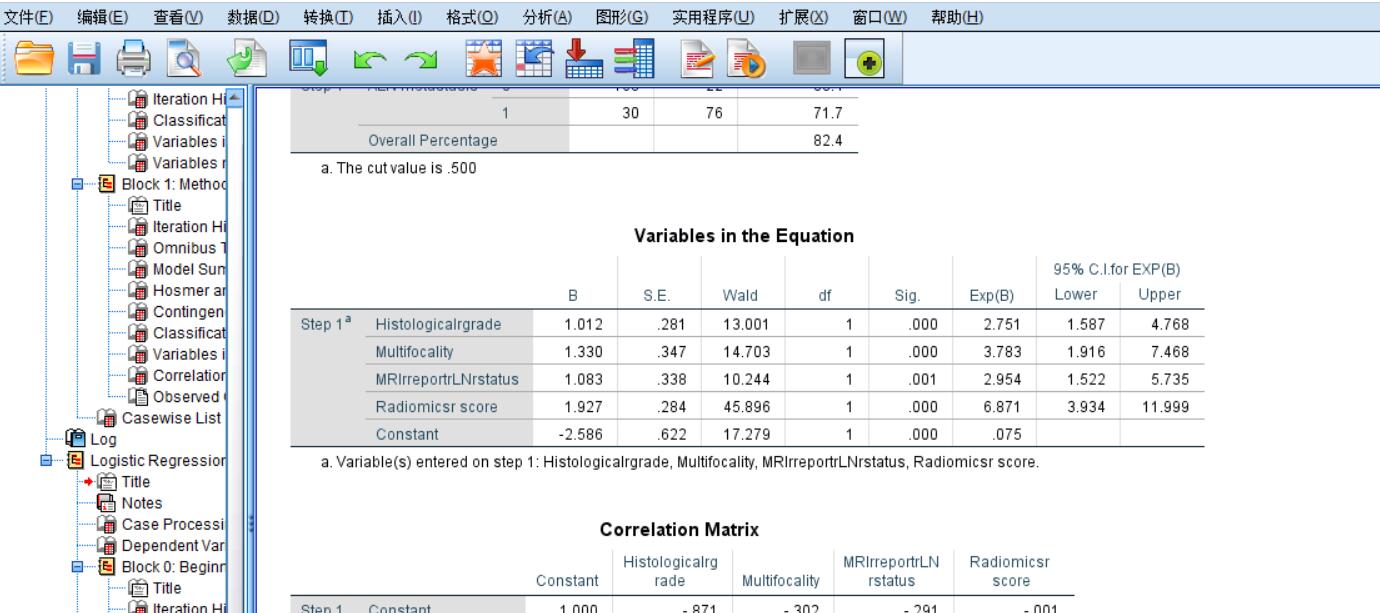


**Nomogram and calibration curve**

code

data1 <- read.csv('E:\\desk\\nomogram.validation.csv')

model1 <- glm(CLASS~Histologicalrgrade+Multifocality+MRIrreportrLNrstatus+Radiomicsrscore,data = data1,family = binomial())

summary(model1)

exp(coef(model1))#计算OR值和可信区间

exp(confint(model1))

ddist <- datadist(data1)

options(datadist = 'ddist')

model2 <- lrm(CLASS~Histologicalrgrade+Multifocality+MRIrreportrLNrstatus+Radiomicsrscore,data = data1)

nom <- nomogram(model2,fun = plogis,funlabel = "Lymph Node Metastasis Risk",lp = F)

plot(nom)

model3 <- lrm(CLASS~Histologicalrgrade+Multifocality+MRIrreportrLNrstatus+Radiomicsrscore,x = T,y = T,data = data1)

cal <- rms::calibrate(model3,method = "boot", B = 1000,bw = T,rule = 'p',sls = 0.05)

plot(cal,main = "Calibration Curve")


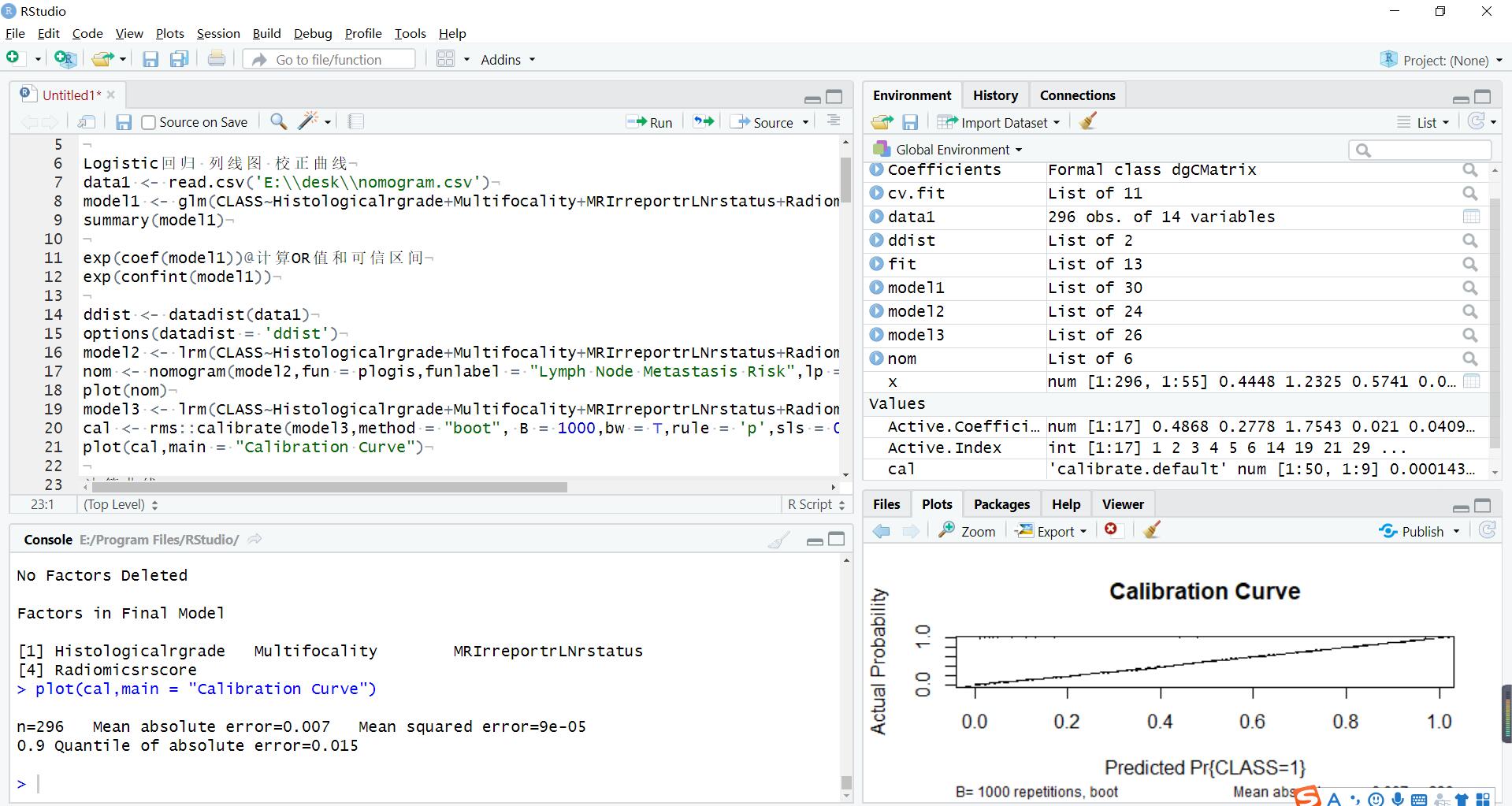


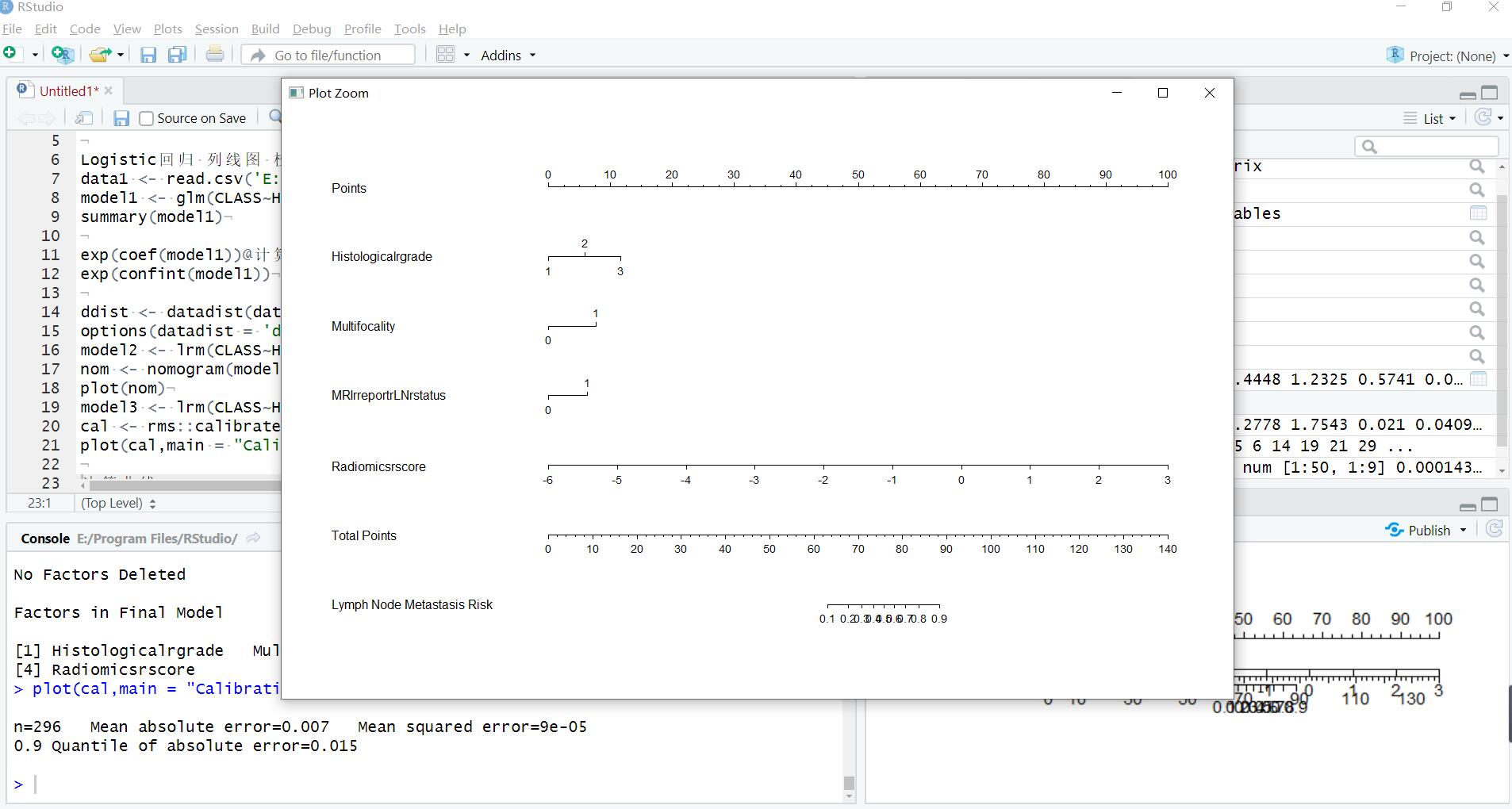


**Training cohort calibration curve**


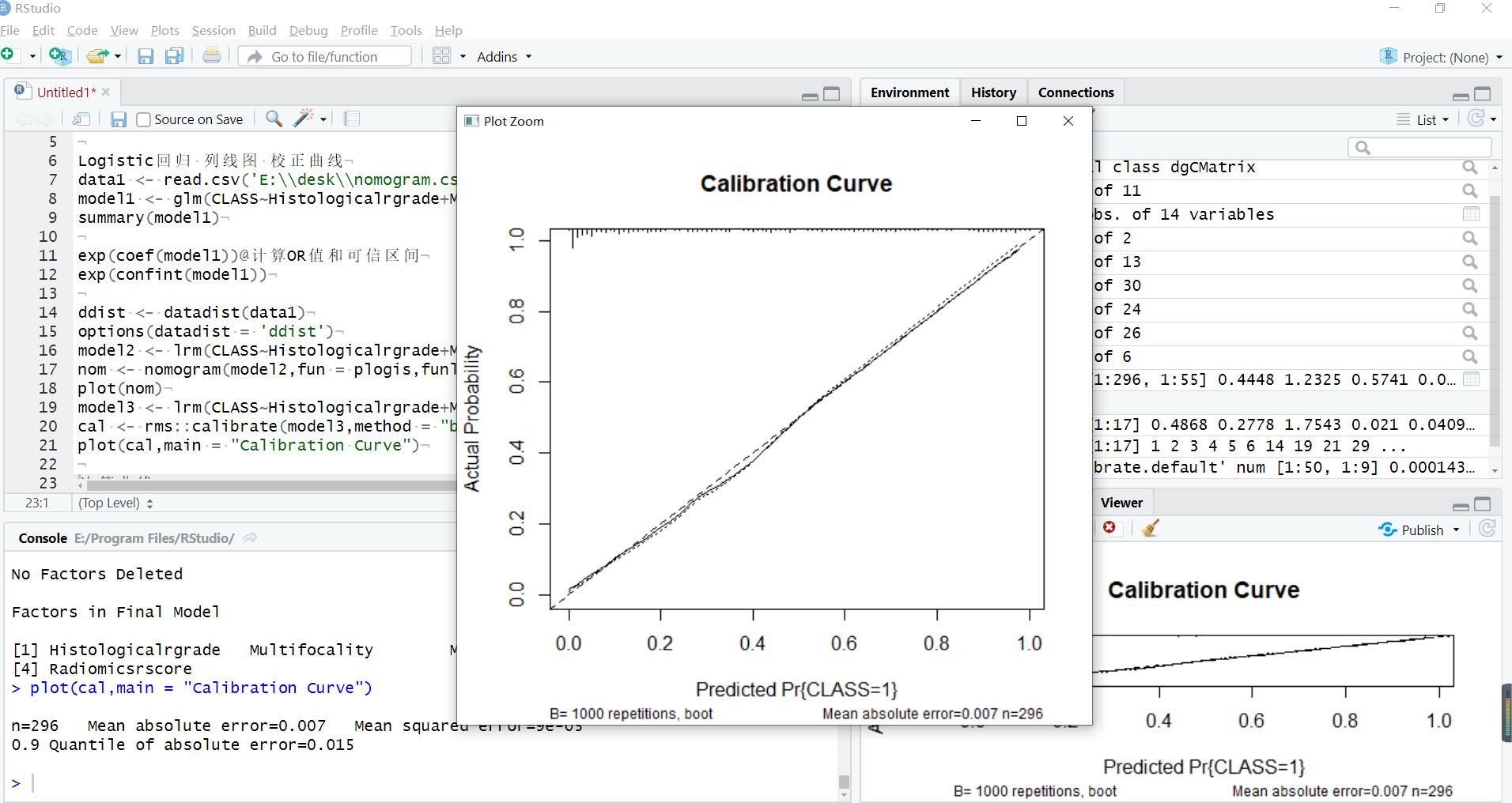


**Validation cohort calibration curve**


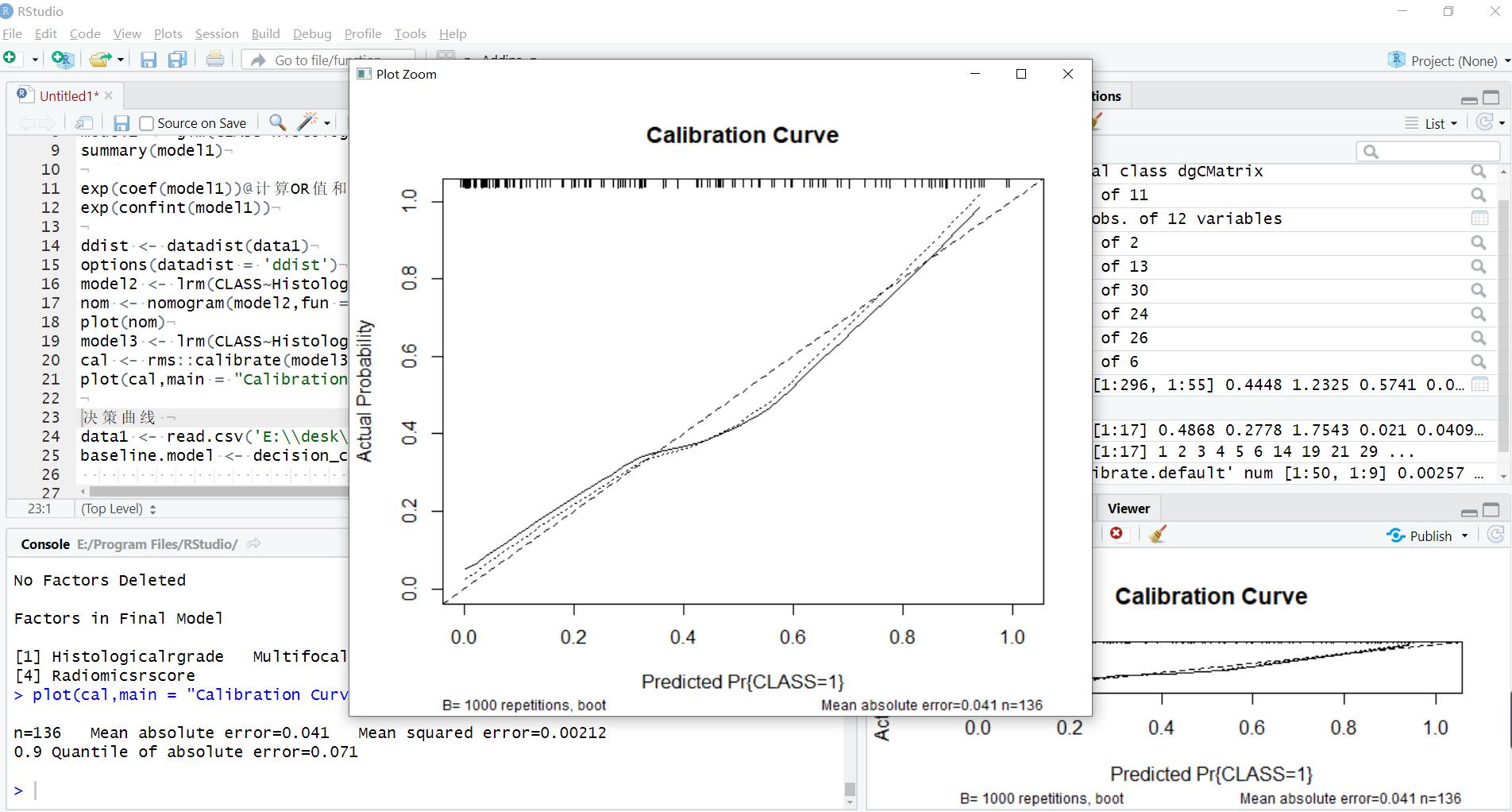


**Import results**

data1 <- read.csv('E:\\desk\\nomogram.csv')

> model1 <- glm(CLASS~Histologicalrgrade+Multifocality+MRIrreportrLNrstatus+Radiomicsrscore,data = data1,family = binomial())

> summary(model1)

Call:

glm(formula = CLASS ~ Histologicalrgrade + Multifocality + MRIrreportrLNrstatus +

Radiomicsrscore, family = binomial(), data = data1)

Deviance Residuals:

Min 1Q Median 3Q Max

-2.3203 -0.5768 -0.1813 0.5018 2.9337

Coefficients:

Estimate Std. Error z value Pr(>|z|)

(Intercept) -2.5861 0.6221 -4.157 3.23e-05 ***

Histologicalrgrade 1.0119 0.2806 3.606 0.000311 ***

Multifocality 1.3305 0.3470 3.835 0.000126 ***

MRIrreportrLNrstatus 1.0832 0.3384 3.201 0.001371 **

Radiomicsrscore 1.9273 0.2845 6.775 1.25e-11 ***

---

Signif. codes: 0 ‘***’ 0.001 ‘**’ 0.01 ‘*’ 0.05 ‘.’ 0.1 ‘ ’ 1

(Dispersion parameter for binomial family taken to be 1)

Null deviance: 386.17 on 295 degrees of freedom

Residual deviance: 226.96 on 291 degrees of freedom

AIC: 236.96

Number of Fisher Scoring iterations: 6

> exp(coef(model1))@计算OR值和可信区间

> exp(confint(model1))

Waiting for profiling to be done...

2.5 % 97.5 %

(Intercept) 0.02108816 0.2444555

Histologicalrgrade 1.61388633 4.8771587

Multifocality 1.94070713 7.6013337

MRIrreportrLNrstatus 1.53696003 5.8209260

Radiomicsrscore 4.12060195 12.6172144

> ddist <- datadist(data1)

> options(datadist = 'ddist')

> model2 <- lrm(CLASS~Histologicalrgrade+Multifocality+MRIrreportrLNrstatus+Radiomicsrscore,data = data1)

> nom <- nomogram(model2,fun = plogis,funlabel = "Lymph Node Metastasis Risk",lp = F)

> plot(nom)

> model3 <- lrm(CLASS~Histologicalrgrade+Multifocality+MRIrreportrLNrstatus+Radiomicsrscore,x = T,y = T,data = data1)

> cal <- rms::calibrate(model3,method = "boot", B = 1000,bw = T,rule = 'p',sls = 0.05)# 做不出图可删除B后面的命令。

Backwards Step-down - Original Model

No Factors Deleted

Factors in Final Model

[1] Histologicalrgrade Multifocality MRIrreportrLNrstatus

[4] Radiomicsrscore

> plot(cal,main = "Calibration Curve")

**ROC curve**

Software MedCalc

**Decision curve**

Software :R 3.6.2

Code

data1 <- read.csv('E:\\desk\\nomogram.csv')

baseline.model <- decision_curve(CLASS~Histologicalrgrade+Multifocality+MRIrreportrLNrstatus+Radiomicsrscore,

data = data1,

thresholds = seq(0,1, by = .005),

bootstraps = 10)

plot_decision_curve(baseline.model,curve.names = "baseline model")

set.seed(123)

plot_decision_curve( list(baseline.model),

curve.names = c("Baseline model"),

col = c("blue"),

lty = c(1),

lwd = c(1),

legend.position = "bottomright")

plot_decision_curve( list(baseline.model),

curve.names = c("Baseline model"),

col = c("blue"),

confidence.intervals = FALSE, #remove confidence intervals

cost.benefit.axis = FALSE, #remove cost benefit axis

legend.position = "none") #remove the legend


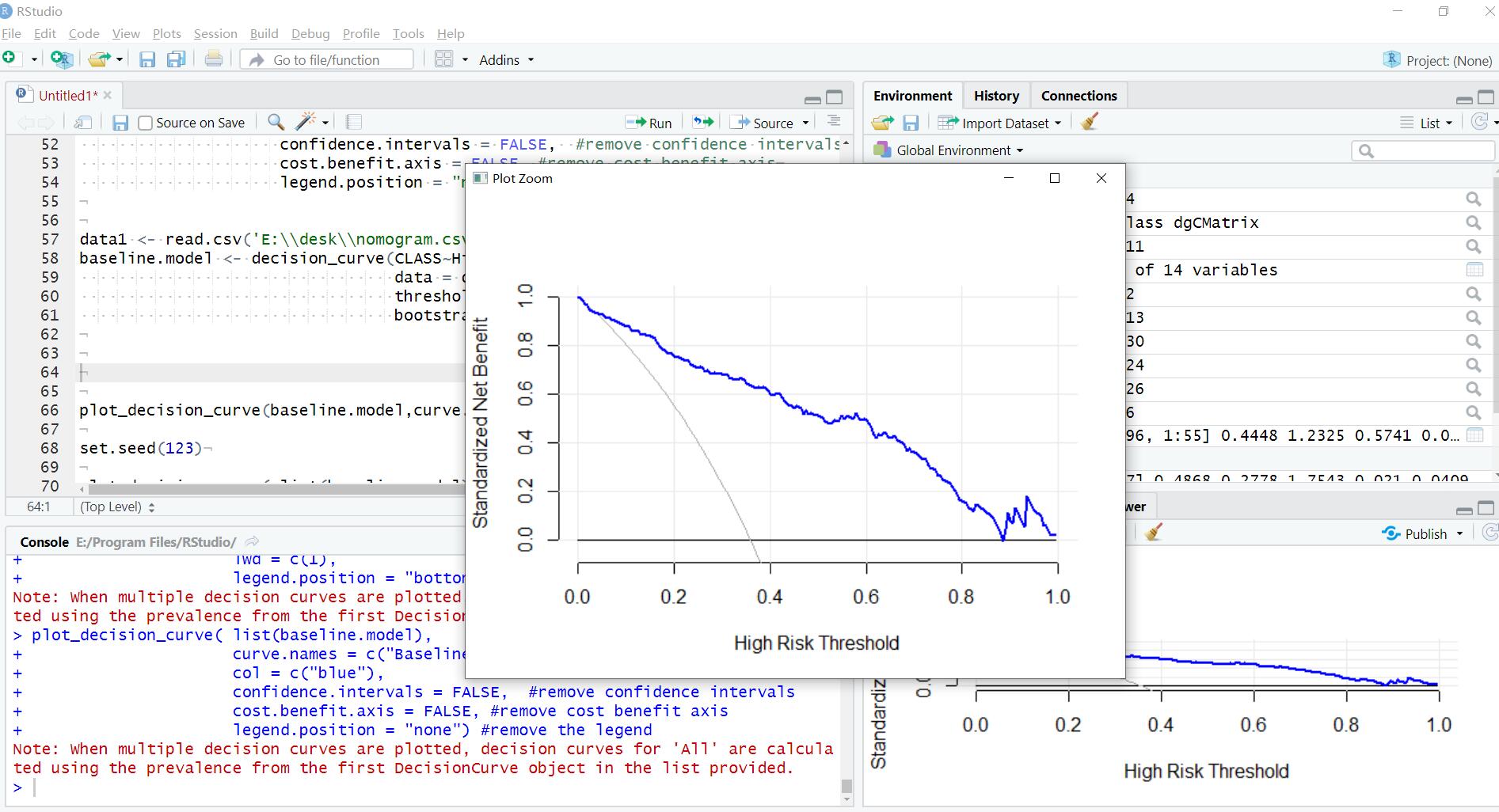


**All the data are in the supplementary materials. All pictures are redrawn using Photoshop.**

**if you encounter any problems using the code, please contact me.**

**Deling Song**

**Email: s312016581@163.com**
